# Trichostatin A Promotes Cytotoxicity of Cisplatin, as Evidenced by Enhanced Apoptosis/Cell Death Markers

**DOI:** 10.3390/molecules29112623

**Published:** 2024-06-03

**Authors:** Yang Zhou, Qun Luo, Fangang Zeng, Xingkai Liu, Juanjuan Han, Liangzhen Gu, Xiao Tian, Yanyan Zhang, Yao Zhao, Fuyi Wang

**Affiliations:** 1 Beijing National Laboratory for Molecular Sciences, CAS Research/Education Center for Excellence in Molecular Sciences, CAS Key Laboratory of Analytical Chemistry for Living Biosystems, Institute of Chemistry, Chinese Academy of Sciences, Beijing 100190, China; zhouyang14@mails.ucas.ac.cn (Y.Z.); qunluo@iccas.ac.cn (Q.L.); hjuan@iccas.ac.cn (J.H.); guliangzhen@iccas.ac.cn (L.G.); tianxiaodeyouxiang@163.com (X.T.); zhangyy0816@iccas.ac.cn (Y.Z.); 2 University of Chinese Academy of Sciences, Beijing 100049, China; 3 School of Environment of Natural Resources, Remin University of China, Beijing 100875, China; zengfg@ruc.edu.cn; 4 National Centre for Mass Spectrometry in Beijing, Beijing 100190, China; 5 College of Traditional Chinese Medicine, Shandong University of Traditional Chinese Medicine, Jinan 250355, China

**Keywords:** trichostatin A, cisplatin, histone acetylation, quantitative proteomics, cytotoxicity

## Abstract

Trichostatin A (TSA), a histone deacetylase (HDAC) inhibitor, promotes the cytotoxicity of the genotoxic anticancer drug cisplatin, yet the underlying mechanism remains poorly understood. Herein, we revealed that TSA at a low concentration (1 μM) promoted the cisplatin-induced activation of caspase-3/6, which, in turn, increased the level of cleaved PARP1 and degraded lamin A&C, leading to more cisplatin-induced apoptosis and G2/M phase arrest of A549 cancer cells. Both ICP-MS and ToF-SIMS measurements demonstrated a significant increase in DNA-bound platinum in A549 cells in the presence of TSA, which was attributable to TSA-induced increase in the accessibility of genomic DNA to cisplatin attacking. The global quantitative proteomics results further showed that in the presence of TSA, cisplatin activated INF signaling to upregulate STAT1 and SAMHD1 to increase cisplatin sensitivity and downregulated ICAM1 and CD44 to reduce cell migration, synergistically promoting cisplatin cytotoxicity. Furthermore, in the presence of TSA, cisplatin downregulated TFAM and SLC3A2 to enhance cisplatin-induced ferroptosis, also contributing to the promotion of cisplatin cytotoxicity. Importantly, our posttranslational modification data indicated that acetylation at H4K8 played a dominant role in promoting cisplatin cytotoxicity. These findings provide novel insights into better understanding the principle of combining chemotherapy of genotoxic drugs and HDAC inhibitors for the treatment of cancers.

## 1. Introduction

**Figure 1:**
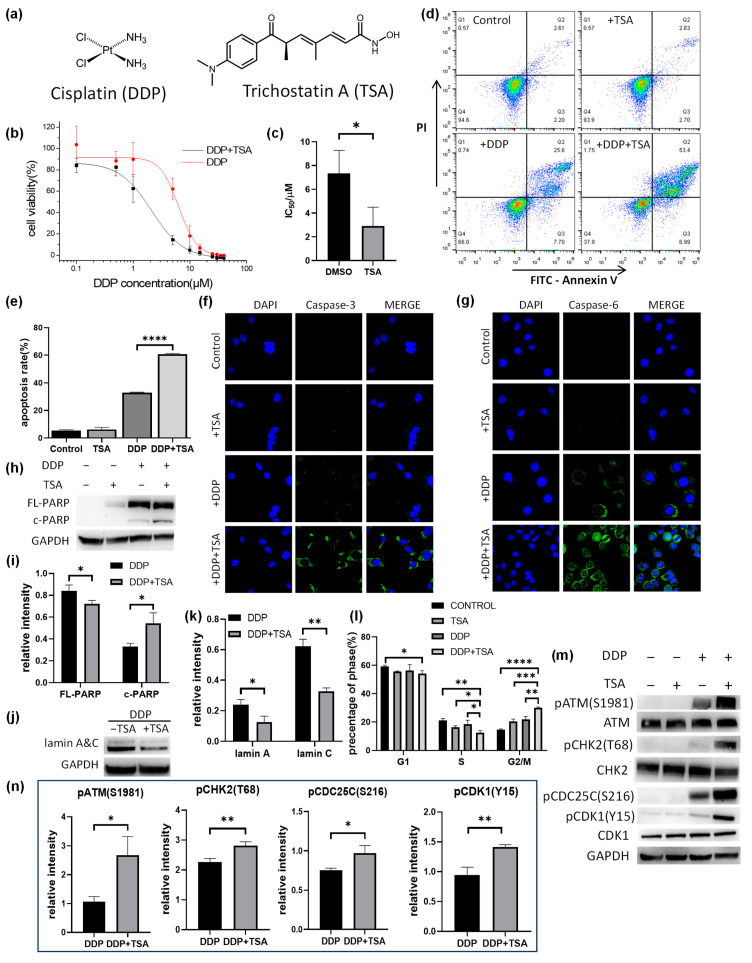
Effect of trichostatin A (TSA) on the cytotoxicity of cisplatin (DDP). (**a**) Structure of DDP and TSA. (**b**,**c**) IC_50_ curves (**b**) and IC_50_ values (**c**) of DDP for A549 cells in the presence (black line) or absence (red line) of TSA (1 μM) (n = 3). (**d**) Apoptosis assays for A549 cells exposed to A549 cells treated with cisplatin (8 μM), TSA (1 μM), or both DDP and TSA. (**e**) Percentage of late stage of apoptotic A549 cells treated under different conditions (n = 3). (**f**,**g**) Immunofluorescence images showing the expression level of cleaved-caspase 3 (**f**) and cleaved-caspase 6 (**g**) in A549 cells treated under different conditions. (**h**) Western blotting data showing the changes in the level of full-length (FL-) and cleaved (c-)PARP in A549 cells treated under different conditions. GAPDH was used as an internal reference. (**i**) Relative abundance to GAPDH of FL-PARP/c-PARP measured by Western blot assay (n = 3). (**j**) Western blotting data showing the changes in the level of lamin A&C in A549 cells treated under different conditions. GAPDH was used as an internal reference. (**k**) Relative abundance to GAPDH of lamin A&C measured by Western blot assay (n = 3). (**l**) Histograms of population distribution at different cell cycles of A549 cells treated under different conditions (n = 3). (**m**,**n**) Western blotting data showing the changes in the level of phosphorylated proteins involved in the ATM-CHK2-CDC25C-CDK1 pathway. GAPDH was used as an internal reference to calculate the relative abundance of the phosphorylated proteins (n = 3). In the panels (**c**,**e**,**i**–**k**,**m**), the data are presented as means ± SD; * *p* < 0.05, ** *p* < 0.01, *** *p* < 0.001, and **** *p* < 0.0001 (Student’s *t* test).

Cisplatin (cis-diamminedichloroplatinum (II), DDP, Figure 1a), one of the most effective antitumor agents, is a small and simple molecule composed of one platinum atom linked to two ammines and two chlorides, of which the primary biological target is genomic DNA [1]. It crosslinks the purine bases on the DNA, causing DNA lesions and subsequently inducing cell cycle arrest and the apoptosis of cancer cells [2]. Cisplatin modification produces distinct changes in the architecture of the DNA duplex and stimulates intercellular responses including DNA repair [1,2]. It is well established in vitro that the high-mobility group box 1 protein (HMGB1) recognizes and binds tightly to 1,2-cisplatin-crosslinked DNA adducts, perhaps preventing the nucleotide excision repair (NER) of cisplatin-induced DNA lesions [1,2,3,4]. Recently, we developed a correlated optical and secondary ion mass spectrometry imaging method to investigate in situ the recognition and interaction between HMGB1 and platinated DNA in single cells [5]. We discovered that cisplatin-crosslinking DNA recruited HMGB1 binding to DNA but impaired interaction between Samd3 and DNA, which disabled the transcription activity of Smad3 [5].

The structure and assembly of genomic DNA have an important impact on the binding affinity of genotoxic drugs like cisplatin. In eukaryotes, DNA in chromatin is organized in arrays of nucleosomes [6], and two copies of each histone protein (H2A, H2B, H3, and H4) are assembled into an octamer that has 146 base pairs of DNA wrapped around it to form a nucleosome core [7]. Nucleosomes play a crucial role as the principal packaging element of genomic DNA, being a determinant of DNA accessibility to endogenous and exogenous molecules [8]. Histones have a flexible and charged NH_2_-terminus (histone tail) that protrudes from the nucleosome, and its modifications like methylation and acetylation could alter chromatin architecture [9]. Acetylation always happens on lysine residues and could neutralize the positive charge of lysine, such that the interaction of histones with negatively charged DNA would be weakened, leading to an increase in DNA accessibility for chemotherapeutic drugs such as cisplatin [8].

The level of histone acetylation is determined by the activities of histone acetyltransferases (HATs) and histone deacetylases (HDACs), which reversibly regulate histone acetylation [10]. Thus, inhibiting HDACs might increase the level of acetylation of histones, promoting the activity of genotoxic anticancer drugs like cisplatin by increasing the accessibility of DNA. Trichostatin A (TSA) is a hydroxamate-type inhibitor of histone deacetylases (Figure 1a) and can elevate the level of histone acetylation [11,12]. It has been reported that the combined use of cisplatin with TSA promoted the cytotoxicity of cisplatin [13,14]. It has been commonly accepted that TSA and its analog suberoylanilide hydroxamic acid (SAHA) promote the acetylation level of histones, causing relaxation of the chromatin structure and increasing genomic DNA attack by cisplatin [14,15]. However, the underlying mechanisms, such as how the acetylation of histones alters the action pathways of cisplatin to enhance its cytotoxicity and the acetylation of which histone(s) and the acetylation of which sites in the histone(s) most contribute to promoting cisplatin cytotoxicity, have been poorly understood.

In the present work, we applied mass spectrometry (MS)-based quantitative proteomics in combination with conventional biochemistry assays and bioinformatics analysis to decipher the correlation between histone acetylation and cisplatin cytotoxicity against human A549 lung cancer cells. We demonstrated that TSA indeed promoted apoptosis and cell cycle arrest at the G2/M phase induced by cisplatin, evidenced by the increase in DNA-bound platinum and the expression level of γH2AX, a well-recognized biomarker of DNA lesions in cells [16]. Quantitative proteomics revealed the presence of TSA-elevated H4K8 acetylation, positively correlating with Pt-DNA binding and DNA lesions. We also found that the presence of TSA resulted in changes in the expression of a number of proteins that regulated signaling pathways such as cell proliferation, apoptosis, ferroptosis, and the migration of cells, contributing to the promotion of cisplatin cytotoxicity against A549 cells.

## 2. Results

### 2.1. TSA Promoted Cisplatin-Induced Apoptosis and Arrest at the G2/M Phase of A549 Cells

Trichostatin A (TSA, Figure 1a) is a well-known deacetylase inhibitor [11,12], showing moderate cytotoxicity with a half-maximal inhibitory concentration (IC_50_) of 36.4 ± 3.5 μM against the human A549 non-small-cell cancer cell line (Figure S1 in the Supporting Information). To confirm the promotion of TSA of the cytotoxicity of cisplatin (Figure 1a), we measured the IC_50_ value of cisplatin for A549 cells in the presence of a low concentration (1 μM) of TSA to exclude the cytotoxicity of A549 cells caused by TSA itself. The IC_50_ value of cisplatin in the presence of 1 μM TSA was 2.28 ± 0.42 μM, significantly lower than that in the absence of TSA (7.81 ± 0.62 μM, Figure 1b,c), indicating that TSA indeed enhances the cytotoxicity of cisplatin. We also measured the IC_50_ values of cisplatin against other human cancer cell lines, including the cisplatin-resistant A549 (A549/DDP) cell line, gastrointestinal stromal tumor (GIST) cell line, HeLa ovarian cell line, HepG2 hepatoma cell line, and MCF-7 breast cancer cell line, in the absence or presence of 1 μM of TSA. The results (Table 1) indicated that TSA promoted the cytotoxicity of cisplatin against all tested cell lines to different extents, with the maximum enhancement of the cytotoxicity of cisplatin for A549 cell lines. Thus, all following experiments were performed on A549 cell lines.

The flow cytometry assays showed that TSA mainly promoted the late apoptosis of A549 cells induced by cisplatin (Figure 1d,e), indicating that TSA enhanced cisplatin cytotoxicity but did not alter the action pathway of the drug, i.e., inducing apoptosis [1,2]. To verify the promotion of TSA on cisplatin-induced apoptosis, we employed immunofluorescence imaging to characterize the level of activated caspase-3, which is a well-known inducer of apoptosis. We found that the presence of TSA dramatically promoted the activity of caspase-3 in the cells treated with cisplatin, as evidenced by the increase in the immunofluorescence intensity of cleaved (or activated) caspase-3 and the caspase-3 activity measured by the caspase activity assay (Figure 1f and Figure S2a). We also observed a remarkable increase in the expression level of activated caspase-6 and a significant increase in caspase-6 activity (Figure 1g and Figure S2b), as well as an increase in cleaved poly-ADP-ribose polymerase (PARP) and the degradation of lamin A&C (Figure 1h,k and Figure S3) in the cells treated with DDP in the presence of TSA. PARP1 could be activated by DNA double-strand breaks (DSBs) and directly to contact cisplatin-crosslinked DNA in human cells to participate in DNA repair [17,18]. PARP1 can be cleaved by caspase-3, which occurs between Asp214 and Gly215, separating the PARP1 amino-terminal DNA-binding domain from the carboxy-terminal catalytic domain [19]. PARP1 cleavage deactivates its repair ability, facilitating cellular disassembly and ensuring the irreversibility and completion of apoptosis [20]. On the other hand, lamin A&C, a pair of nuclear envelope proteins, also plays an important role in DNA repair, nuclear assembly, and chromatin organization, and it can be degraded by activated caspase-6 [21], resulting in nuclear dysregulation and apoptosis [22,23]. Our results demonstrated that the level of cleaved PARP (c-PARP) was upregulated, whereas the level of lamin A&C in A549 cells was significantly downregulated in A549 cells treated with cisplatin in the presence of TSA, evidencing the TSA-enhanced cisplatin-induced apoptosis of A549 cells by activating the caspase pathway.

We further explored the mechanism by which TSA promotes double-stranded DNA breaks (DSBs) caused by cisplatin, activating the ATM-CHK2-CDK1 signaling pathway and leading to G2/M arrest. Our Western blot assays demonstrated that the phosphorylation of ATM, CHK2, and CDK1 involved in this pathway was significantly elevated in the A549 cells treated with DDP in the presence of TSA (Figure 1m,n). This indicated that in the presence of TSA, cisplatin caused more DNA DSBs to activate the ATM-CHK2-CDK1 signaling pathway, leading to the G2/M arrest of cells.

### 2.2. TSA Enhanced Cisplatin Binding to DNA

**Figure 2:**
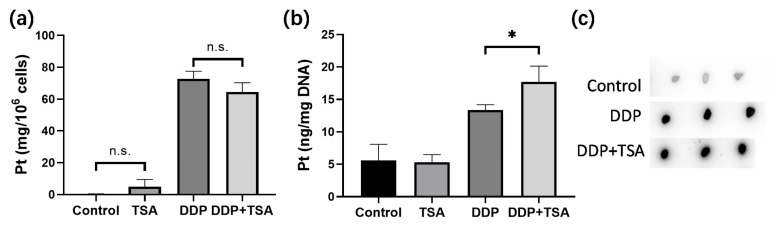

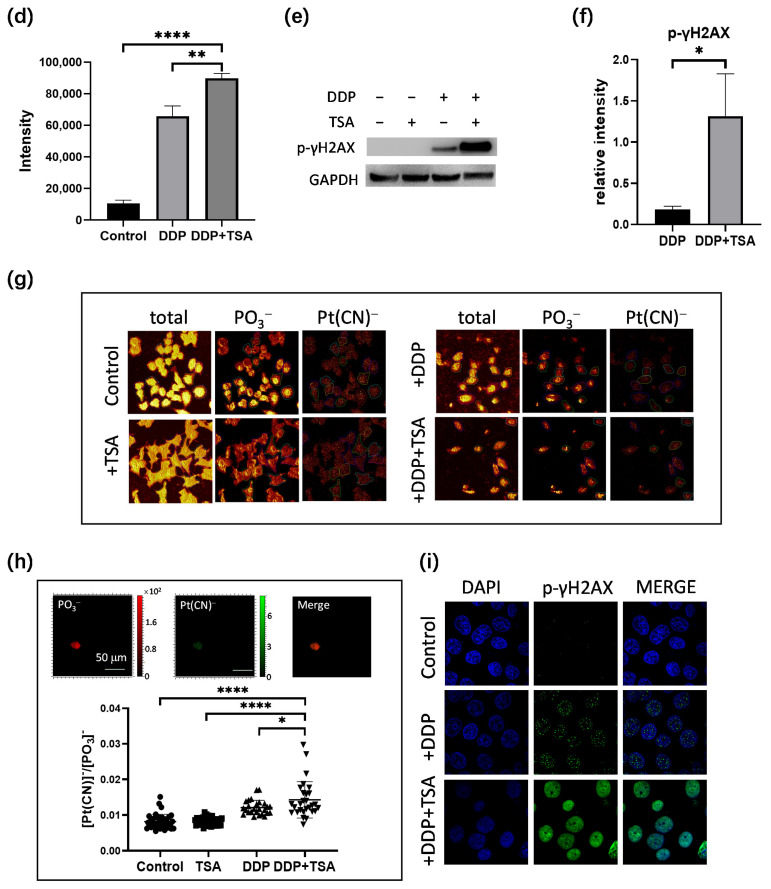
Effects of TSA on cellular uptake and DNA binding of cisplatin. (**a**,**b**) Cellular uptake of cisplatin (**a**) and concentration of DNA-bound cisplatin (**b**) in A549 cells treated with cisplatin (8 μM) in the absence or presence of TSA (1 μM), determined by ICP-MS; the data are presented as ng Pt/10^6^ cells and ng Pt/mg DNA, respectively (n = 3). (**c**,**d**) Dot blotting data showing the changes in the amount of DNA-bound Pt, indicated by the intensity of dots in A549 cells treated under different conditions (n = 3). (**e**,**f**) Western blotting data showing the changes in the expression level of γH2AX in A549 cells treated under different conditions. GAPDH was used as an internal reference to calculate the relative abundance of γH2AX (n = 3). (**g**) ToF-SIMS images in view of 200 × 200 μm showing the accumulation of cisplatin, indicated by the signal intensity of Pt(CN)^−^ ions in nuclei, which were rendered by imaging PO_3_^−^ in A549 cells treated under different conditions. (**h**) The abundance ratio of Pt(CN)^−^ to PO_3_^−^ (the inserts) in each A549 cell shown in (**g**). We randomly selected 20 cells in each group for statistics analysis. (**i**) Immunofluorescence images showing the level of γH2AX in A549 cells treated under different conditions. In the panels (**a**,**b**,**d**,**f**,**h**), the data are presented as means ± SD; n.s. means no significantly, * *p* < 0.05, ** *p* < 0.01, and **** *p* < 0.0001 (Student’s *t* test).

It is well recognized that cisplatin exerts cytotoxicity by attacking DNA to cause lesions on genomic DNA [1,2,5]. To further verify whether TSA promoted the cytotoxicity of cisplatin by enhancing cisplatin-induced DNA lesions, we employed ICP-MS, ToF-SIMS, and immunofluorescence imaging to determine the platinum level on a large scale of cells and in single cells, respectively. Firstly, we cultured A549 cells with 8 μM of cisplatin for 48 h in the absence and presence of TSA (1 μM), respectively. Then, we harvested the cells and measured the cellular accumulated Pt and DNA-bound Pt by ICP-MS. The results showed that TSA did not pronouncedly change the cellular uptake of cisplatin, presented as nanograms of Pt per million cells (Figure 2a), but significantly increased the amount of cisplatin binding to DNA (Figure 2b), which was evidenced by the dot blotting assay (Figure 2c,d), where the cisplatin-crosslinked DNA was stained by a specific antibody of platinated DNA [24]. Moreover, we found that the expression level of phosphorylated γH2AX, which is a well-known biomarker of DNA damage caused by genotoxic drugs like cisplatin [16], significantly increased in the cells treated with DDP and TSA in comparison with the cells treated with cisplatin alone (Figure 2e,f), also evidencing the elevation of cisplatin-bound DNA due to TSA co-administration.

Next, we applied ToF-SIMS signal cell imaging to determine the level of cisplatin in single cells, following the procedure described previously (Figure 2g) [5,25,26]. The results indicated that the accumulation of Pt in nuclei normalized to the signal intensity of imaging PO_3_^−^ ions [5,25,26] of the cells treated with DDP and TSA pronouncedly increased in comparison to that of the cells treated with DDP only (Figure 2h). This means at the single-cell level, TSA increased the level of cisplatin binding to DNA, which was supported by the increase in the immunofluorescence intensity of phosphorylated γH2AX in the single cells (Figure 2i). Collectively, these results demonstrated at both the large scale of cells and the single cell level that TSA promoted the cytotoxicity of cisplatin by elevating cisplatin-induced DNA lesions.

### 2.3. Quantitative Proteomics Deciphered the Mechanism of Action of Cisplatin in the Absence and Presence of TSA

**Figure 3:**
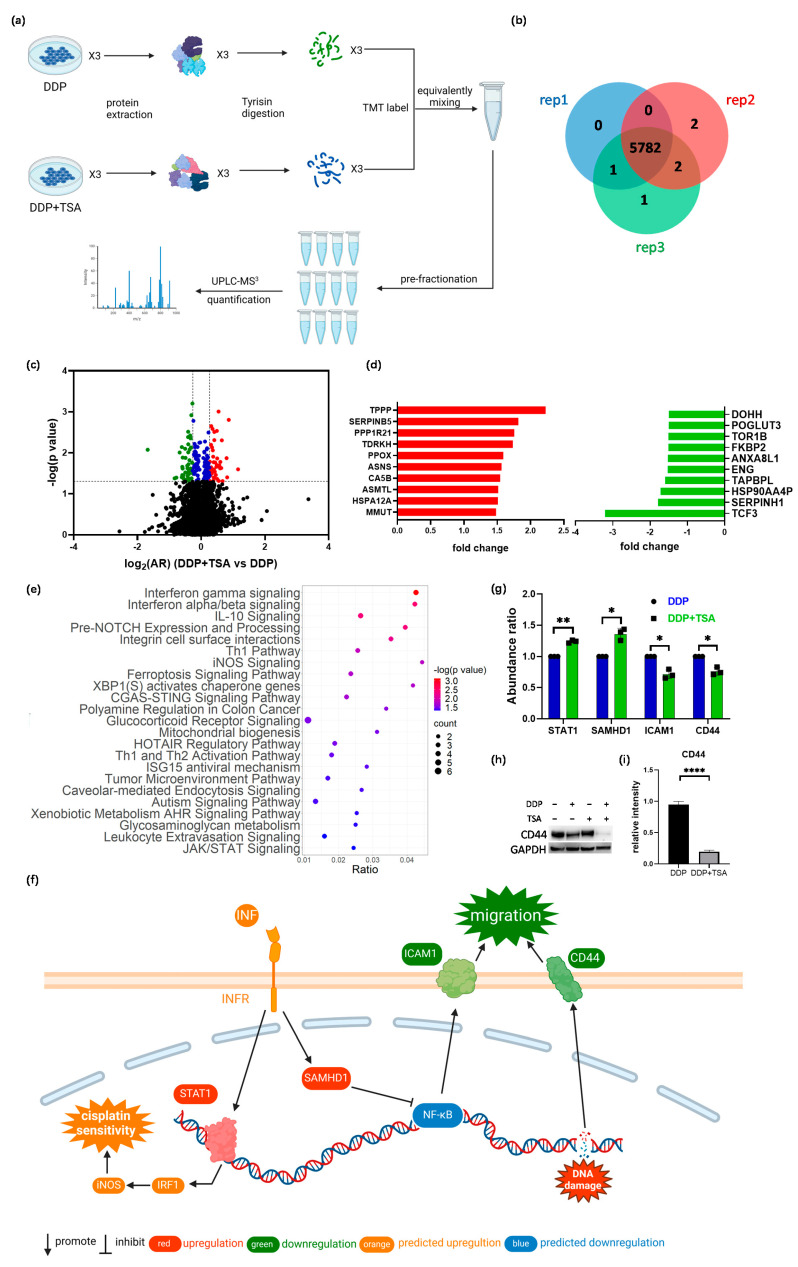
MS-based quantitative proteomics identified differentially expressed proteins. (**a**) Workflow of quantitative proteomics analysis. (**b**,**c**) Venn diagram (**b**) and volcanic map (**c**) of proteins identified in A549 cells treated with 8 μM DDP in the presence or absence of 1 μM TSA for 48 h. In the volcanic map (**c**), black points refer to proteins with a *p*-value of > 0.05 (i.e., −lg *p* < 1.3), while red, blue, and green points refer to proteins with a *p*-value ≤ 0.05 and a log_2_(FC) (DDP + TSA vs. DDP) of ≥ 0.26 (red points), a log_2_(AR) of <0.26 and >−0.26 (blue points), or a log_2_(AR) of ≤−0.26 (green points). (**d**) Top 10 upregulated (red) and downregulated (green) proteins identified in the cells treated with DDP and TSA compared with those in the cells treated with DDP alone. (**e**) IPA enriched canonical signaling pathways with which all differentially expressed proteins (DEPs) with |FC| > 1.2 identified in A549 cells treated with DDP and TSA, compared with the proteins expressed in A549 cells treated with DDP only, are associated. (**f**) Diagrammatic representation of interferon-gamma (INF-γ) signaling pathway. (**g**) Histogram of the abundance ratio of selected DEPs expressed in A549 cells treated with DDP and TSA compared with the abundance of the same proteins expressed in the cells treated with DDP only. (**h**,**i**) Western blotting data showing the changes in the level of CD44 expressed in A549 cells treated under different conditions. GAPDH was used as an internal reference to calculate the relative abundance of CD44 (n = 3). The data in (**g**,**i**) are presented as means ± SD; * *p* < 0.05, ** *p* < 0.01, and **** *p* < 0.0001 (Student’s *t* test).

In order to further delineate the underlying molecular mechanism by which TSA promoted the cytotoxicity of cisplatin, we applied mass spectrometry (MS)-based quantitative proteomics analysis coupled with tandem-mass-tag (TMT) labeling to profile the expression of proteins in A549 cells exposed to 8 μM of cisplatin and 1 μM of TSA (designated as DDP + TSA group) in comparison with that of A549 cells treated with 8 μM of cisplatin only (designated as DDP group, Figure 3a). We identified 5782 proteins that were commonly expressed in both groups of cells in three independent biological replicates (Figure 3b). There were 195 proteins identified with a statistical significance (*p* < 0.05) (Table S1 in the Supporting Information); among them were 48 proteins upregulated with an abundance ratio (AR) of >1.20 and 47 proteins downregulated with an AR < 0.83 in the A549 cells treated with DDP and TSA compared with those expressed in A549 cells treated with DDP alone (Figure 3c, Table S2). Notably, as shown in Figure 3d, the tubulin polymerization promoting protein (TPPP or p25, fold-change (FC) = 2.23) and transcription factor 3 (TCF3, FC = −3.21), where FC is equal to AR when the AR of a protein is >1.0 but the negative to the reciprocal of AR when the AR of a protein is <1.0, were mostly upregulated and downregulated, respectively. TPPP is a microtubule-associated protein that polymerizes tubulin into normal or aberrant microtubules, depending on its concentration and phosphorylation state [27]. The low TPPP expression indicated a poor prognosis in hepatocellular carcinoma [28]. Therefore, the elevated TPPP level in A549 cells treated with cisplatin in the presence of TSA might be associated with higher cytotoxicity of cisplatin. On the other hand, TCF3, also named transcription factor E2-alpha (E2A), is a multifunctional BHLH (basic helix loop helix) transcription factor and plays a crucial role in the development and differentiation of lymphocytes, being correlated with the development of lymphoma and leukemia [29]. The increased expression of TCF3 could enhance the proliferation, invasion, and metastasis of breast cancer cells and was associated with poor prognosis [30]. Hence, the reduction in the expression of TCF3 in A549 cells may also contribute to the promotion of cisplatin cytotoxicity by TSA.

To sort out the pharmacological functions of all other DEPs with an |FC| > 1.2 expressed in A549 cells treated with cisplatin and TSA in comparison with those expressed in A549 cells treated with DDP alone (Table S2), we used Ingenuity Pathways Analysis (IPA) to enrich the canonical signaling pathways which the DEPs were associated with. The results revealed that the DEPs were most associated with interferon (INF)-γ and -α/β signaling pathways (Figure 3e) in which the transcription factor signal transducer and activator of transcription 1 (STAT1, FC = 1.24) and a deoxynucleotide triphosphate (dNTP) hydrolase sterile alpha motif and histidine-aspartate domain-containing protein 1 (SAMHD1, FC = 1.39) were upregulated (Figure 3f,g). INF-γ/α/β signaling plays an important role in modulating immune responses, and its activation could enhance the efficacy of cisplatin [31,32]. STAT1 is a crucial mediator of IFN signaling and is considered a tumor suppressor. The activation or overexpression of STAT1 could upregulate iNOS signaling pathways (Figure 3f) by promoting interferon regulatory factor 1 (IRF1) expression, resulting in the induction of inducible nitric oxide synthase (iNOS or NOS2), which, in turn, improved the effect of cisplatin on cancer cells [33]. On the other hand, SAMHD1 can suppress NF-κB activation subjected to interferon stimulation [34], which, as a consequence, increased the expression of the intercellular adhesion molecule 1 (ICAM1) [35]. Our global proteomics analysis demonstrated that the expression of ICAM1 was downregulated with an FC of −1.43 in the cells treated with TSA and DDP in comparison with the protein in the cells treated with DDP only (Figure 3g). Another protein involved in regulating the tumor microenvironment pathway is CD44 [36], of which the expression in A549 cells treated with DDP and TSA was also downregulated (FC = −1.35) (Figure 3f,g). To verify that the downregulation of both ICAM1 and CD44 reduces the migration of A549 cells by regulating the microenvironment of cells, we performed a wound-healing experiment. The results showed that after growth of 36 h in TSA and DDP-free medium, the A549 cell previously treated with DDP and TSA had a slower recovery rate compared with the cells pretreated with DDP or TSA alone (Figure S5). These data collectively indicate that the addition of TSA led to the upregulation of STAT1 and SAMHD1 and the downregulation of ICAM1 and CD44, enhancing cisplatin sensitivity and reducing cell migration [37], respectively, as shown in Figure 3f.

**Figure 4:**
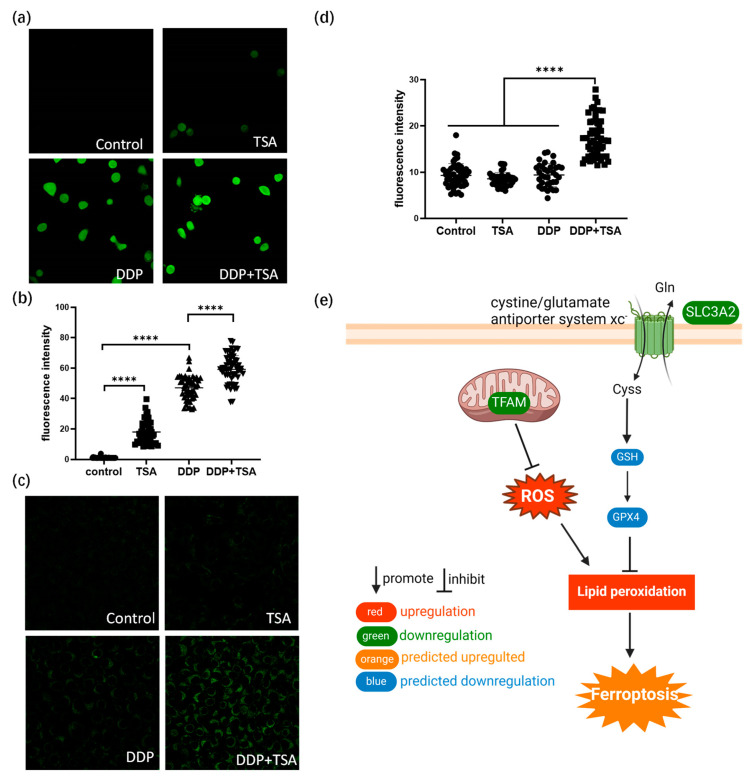
TSA enhances ferroptosis induced by cisplatin. (**a**) Fluorescence images of ROS probe DCFH-DA showing the ROS level of A549 cells treated with DDP (8 μM), TSA (1 μM), or DDP (8 μM) and TSA (1 μM) for 48 h. (**b**) ROS level in A549 cells measured by the fluorescence intensity of randomly selected single cells shown in (**a**). (**c**) Fluorescence images of liperfluo probe showing the level of lipid peroxidation in A549 cells treated under different conditions. (**d**) Lipid peroxidation level in A549 cells measured by the fluorescence intensity of randomly selected single cells shown in (**c**). (**e**) Diagrammatic presentation of ferroptosis pathway induced by SLC3A2 and TFAM. In panels (**b**,**c**), the data are presented as means ± SD; and **** *p* < 0.0001 (Student’s *t* test).

The ferroptosis signaling pathway was also enriched by IPA to be a core signaling pathway highly associated with the DEPs identified in A549 cells treated with DDP and TSA (Figure 3e). This was evidenced by the significantly elevated levels of reactive oxygen species (ROS) and lipid peroxidation (Figure 4a–d), both of which are markers of ferroptosis [38]. It is notable that among the proteins involved in the ferroptosis signaling pathway, Solute Carrier Family 3 Member 2 (SLC3A2) and mitochondrial transcription factor A (TFAM) were downregulated by 1.2- and 1.32-fold, respectively, in the cells exposed to DDP and TSA, compared with the proteins expressed in the cells treated with DDP alone (Table S2). INF-γ could reduce the expression of SLC3A2, a subunit of cystine/glutamate antiporter system xc^−^, and the low expression of system xc^−^ impaired the cysteine metabolism [39,40]. As cysteine is a precursor for reduced glutathione (GSH) biosynthesis; deficiency in GSH biosynthesis results in inactivating the enzyme GPX4, which uses GSH to eliminate lipid peroxides [41,42]. Hence, the underexpression of SLC3A2 enhances lipid peroxidation, inducing ferroptosis. Moreover, TFAM is involved in the mitochondrial biogenesis pathway, and its downregulation increased reactive oxygen species (ROS), also inducing ferroptosis by elevating lipid peroxidation [43]. To confirm that cisplatin could induce ferroptosis in the presence of TSA, we used DCFH-DA and liperfluo probes, which are specific fluorescence probes for imaging ROS and lipid peroxidation, respectively, in cells [39,44], to detect the level of ROS and lipid peroxidation in A549 cells. The results indicated that the levels of ROS and lipid peroxidation were significantly increased when cells were exposed to DDP in the presence of TSA (Figure 4e), evidencing that TSA promoted cisplatin cytotoxicity also by enhancing ferroptosis. Furthermore, we used a ferroptosis inhibitor, Ferrostatin-1, to pre-treat A549 cells prior to exposing the cells to DDP and TSA; the results showed that the cytotoxicity of DDP in the presence of TSA was reduced (Figure S6). This again supports that the TSA promotion of cisplatin cytotoxicity is partially attributable to the enhancement of ferroptosis.

**Figure 5:**
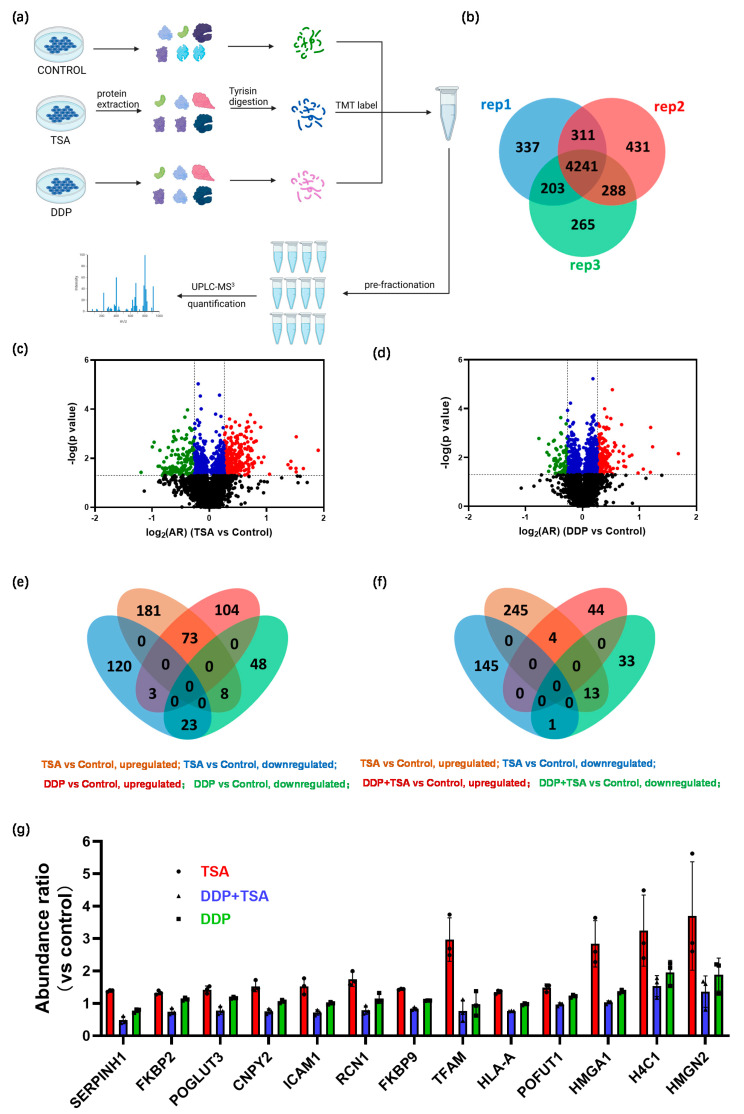
MS-based quantitative proteomics identified differentially expressed proteins. (**a**) Workflow of quantitative proteomics analysis. (**b**–**d**) Venn diagram (**b**) and volcanic maps (**c**,**d**) of proteins identified in A549 cells treated with 1 μM TSA only (**c**) or 8 μM DDP only (**d**) for 48 h. The Venn diagram shows 4241 proteins commonly expressed in three replicated experiments of the two groups of cells. In the volcanic maps, black points refer to proteins with a *p*-value of >0.05 (i.e., −lg *p* < 1.3), while red, blue, and green points refer to proteins with a *p*-value ≤ 0.05 and a log_2_(AR) (TSA or DDP vs. control) of ≥0.26 (red points), a log_2_(AR) of <0.26 and >−0.26 (blue points), or a log_2_(AR) of ≤−0.26 (green points). (**e**) Venn diagram showing differentially expressed proteins (DEPs) with a *p* ≤ 0.05 and a log_2_(AR) (TSA vs. control or DDP vs. control) of ≥ 0.26 or a log_2_(AR) of <−0.26, shown in (**c**). (**f**) Venn diagram showing DEPs with a *p* ≤ 0.05 and a log_2_(AR) (DDP + TSA vs. DDP) of ≥0.26 or a log_2_(AR) of <−0.26, shown in (**d**). (**g**) Histogram of the abundance ratio of selected DEPs expressed in A549 cells treated under different conditions. The abundance of the selected proteins was normalized to that of each selected protein expressed in the control group.

To distinguish whether the DEPs identified in the cells treated with TSA and DDP resulted from the enhanced cytotoxicity of cisplatin by TSA or from the cytotoxicity of TSA itself, we also performed quantitative proteomics analysis similar to that described above to profile the protein expression of A549 cells treated with TSA or DDP only (Figure 5a). We identified 4241 proteins that were commonly expressed in three groups of cells, designated as the control group, TSA group, and DDP group (Figure 5b and Tables S3 and S4), in three replicates. We identified 408 DEPs with a *p*-value < 0.05, among which were 262 proteins upregulated and 146 proteins downregulated with an |FC| > 1.2 in A549 cells treated with 1 μM of TSA only compared with those expressed in the control group of cells (Figure 5c, Table S5). Although TSA displayed only moderate cytotoxicity, with an IC_50_ of 36.37 μM (Figure S1), the proteomics results indicated that even a low concentration (1 μM) of TSA could remarkably disturb the protein profiling of cells. For the A549 cells treated with 8 μM of DDP only, we identified 259 DEPs with a *p*-value < 0.05, of which 180 proteins were upregulated and 79 proteins were downregulated, respectively, compared with those expressed in the control group of cells (Figure 5d, Table S6). Interestingly, 107 DEPs were commonly identified in both groups of A549 cells treated with TSA alone and DDP alone. Among them, 73 proteins were upregulated and 23 proteins were downregulated in both groups of cells (Figure 5e). The IPA enriched canonical signaling pathways, with which all DEPs identified in the TSA group are closely associated, are similar to those with which all DEPs identified in DDP group are closely associated (Figure S7a,b). The commonly regulated signaling pathways by TSA and DDP were mainly involved in eukaryotic translation, EIF2 signaling, oxidative phosphorylation, and sirtuin signaling. This is probably attributable to the fact that both TSA and DDP alter proteome profiles by changing the gene expression, though TSA changes gene expression by altering nucleosome structure [12] while DDP changes protein expression by causing DNA lesions [1,2].

We further compared the DEPs identified in the DDP + TSA group (Table S2) with those found in the TSA-only group (Table S6). Notably, only five proteins were commonly upregulated (four) or downregulated (one) in both groups of A549 cells (Figure 5f). In contrast, more proteins (thirteen) upregulated by TSA were downregulated by DDP in the presence of TSA (Figure 5f,g), but were only downregulated slightly or even upregulated by DDP in the absence of TSA (Figure 5g). The thirteen proteins were shown to be closely associated with INF-γ/α/β signaling, the tumor microenvironment pathway, iNOS signaling, etc. (Figure S7c), similar to those (Figure 3e) enriched by IPA based on all DEPs identified in A549 cells treated with DDP in the presence of TSA (Table S2). This similarity unambiguously indicates that it was the TSA-induced elevation of histone acetylation that increased the accessibility of genomic DNA to cisplatin, enhancing DNA damage by the drug to promote its cytotoxicity.

### 2.4. TSA Elevated the Acetylation of H4K8 to Promote Cisplatin Activity

**Figure 6:**
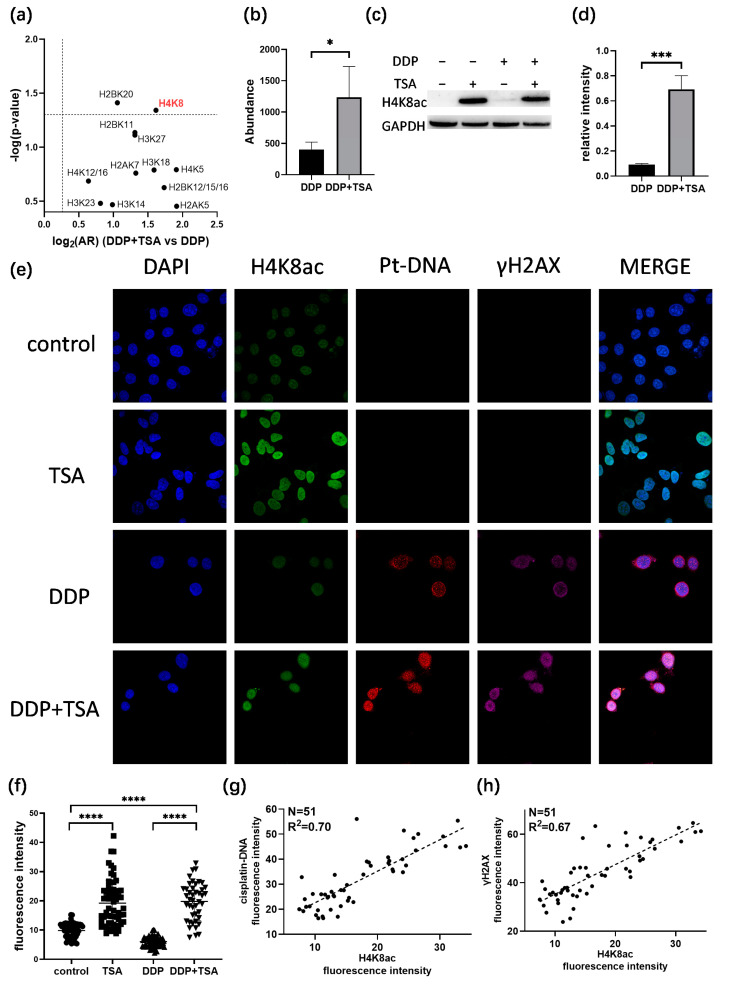
Correlation between histone acetylation and Pt-induced DNA damage. (**a**,**b**) Changes in acetylation level of histone lysine residues (**a**) and H4K8 (**b**) in A549 cells treated with 8 μM DDP and 1 μM TSA (designated DDP + TSA group) in comparison with that of A549 cells treated with 8 μM DDP only, measured by quantitative proteomics analysis (n = 3). (**c**,**d**) Immunoblotting data showing changes in acetylation level of H4K8 in A549 cells treated with 8 μM DDP in the presence (+) or absence (−) of TSA (1 μM) (n = 3). (**e**) Immunofluorescence images showing the levels of H4K8ac, cisplatin-modified DNA (Pt-DNA), and γH2AX in A549 cells treated under different conditions. (**f**) H4K8ac level in A549 cells measured by the fluorescence intensity of randomly selected single cells, shown in (**e**). (**g**) Correlation between the levels of H4K8ac and Pt-DNA, indicated by immunofluorescence signal intensity of H4K8 and Pt-DNA, shown in (**e**). (**h**) Correlation between the levels of H4K8ac and γH2AX, indicated by immunofluorescence signal intensity, shown in (**e**). In panels (**b**,**d**,**f**), the data are presented as means ± SD; * *p* < 0.05, *** *p* < 0.001, and **** *p* < 0.0001 (Student’s *t* test).

Next, we investigated the acetylation of which sites in histones play a crucial role in increasing the accessibility of genomic DNA to DDP attack. To achieve this goal, we examined the acetylation of histones based on the quantitative proteomics data listed in Table S1 for A549 cells treated with 8 μm of cisplatin in the absence and presence of 1 μm of TSA for 48 h. We identified the acetylation of 15 lysine residues in histones 2/3/4 (Table S7), and the acetylation levels were all upregulated in the A549 cells treated with DDP and TSA compared with those in the A549 cell treated with DDP only (Figure 6a, Table S8). However, only the changes in the acetylation level of H4K8 (Figure S8) and H2BK20 were shown to be significant, with an AR > 2.0 and a *p*-value < 0.05 (Figure 6b,c), where the abundance ratio (AR) = (abundance of acetylation at H4K8)_DDP+TSA_/(abundance of acetylation at H4K8)_DDP_. The Western blot assays confirmed the significant change in the acetylation of H4K8 in the cells of the DDP + TSA group compared with that in the cells of the DDP group (Figure 6c,d). Given that H4K8 showed the largest fold-change in acetylation level when subjected to the treatment of DDP and TSA, we used immunofluorescence imaging to measure the acetylation level of H4K8 (Green) in single A549 cells treated under different conditions. The results showed that H4K8 acetylation was indeed significantly increased in the cells treated with TSA or DDP in the presence of TSA (Figure 6e,f). To further verify that the acetylation of H4K8 enhanced DNA damage by DDP, we used immunofluorescence imaging to measure the level of the cisplatin-damaged DNA (Pt-DNA) and the DNA damage biomarker γH2AX, which were illuminated by a fluorophore-conjugated Pt-DNA (Red) antibody and γH2AX antibody (Magenta), respectively, and the level of H4K8ac in the nuclear region rendered by nuclear dye DAPI (Blue) (Figure 6e). Significantly, we randomly selected 51 cells, shown in Figure 6e, to examine the linear correlation of the levels of Pt-DNA and H4K8ac and obtained a squared correlation coefficient of 0.70 (Figure 6g). The levels of γH2AX and H4K8ac also had a positive correlation, with a squared correlation coefficient of 0.67 (Figure 6h). These results again evidenced that H4K8 acetylation plays a dominant role in promoting cisplatin activity.

Acetylation neutralizes the positive charge of lysine residues in histones, weakening charge-dependent interactions between histones and nucleosomal DNA [45]. The amino-terminal tail of histone H4 contains four lysine residues at positions 5, 8, 12, and 16, and the acetylation of the histone H4 tail substantially reduces its affinity to DNA, thus increasing the accessibility of DNA to genotoxic drugs [46]. H4K8ac is associated with telomere attrition, which might activate a DNA damage response, leading to chromosomal instability [47]. γH2AX formed in chromatin around DNA DSBs [48], and our results showed that H4K8ac colocalized with γH2AX, evidencing that the TSA-induced elevation of acetylation at H4K8 enhanced DNA damage by DDP, evidenced by the elevation of γH2AX. However, during DSB repair, the acetylation of H4 is also a key factor in limiting the binding of H4 to the acidic patch, which, as a consequence, creates open and flexible nucleosome structures that favor DNA repair [49]. Relaxed chromatin domains caused by H4 acetylation could load DNA repair proteins onto the chromatin adjacent to the DSBs [50]. Therefore, H4K8ac may facilitate the accumulation of repair molecules, e.g., MDC1 and BRCA1 and KU70 and KU80 to DSB sites, resulting in the homologous recombination (HR) and non-homologous end joining (NHEJ) repair of DSBs [51]. Moreover, the acetylation of the histone H4 tail promoted gene transcription, and BRD4 could bind directly to H4ac, especially to H4K8ac/K12ac, elongating transcription and recruiting transcriptional co-activators to genes associated with DNA repair [52,53]. These may reduce the cytotoxicity of cisplatin due to H4K8 acetylation. However, our study showed that H4K8 acetylation significantly promoted the cytotoxicity of the genotoxic drug cisplatin. This was attributed to the fact that H4K8 acetylation led to more cisplatin-induced DNA lesions, which were beyond the repair capacity, ultimately resulting in an increase in cisplatin-induced cell apoptosis and ferroptosis. 

The acetylation of H4K16 is also a kind of response to DNA damage [54], and H4K16ac could be a marker of chromatin relaxation [55]. H3K14 acetylation induced by UV damage facilitates DNA repair [56,57]. H4K5 acetylation was reported to increase in the chromatin in the vicinity of DSBs caused by γ-rays. The acetylation of H3K18, H3K23, H3K27, and H4K12 has also been thought to act as active enhancers and is associated with gene activation [58,59,60]. Hence, HDAC inhibitors could increase the expression of many tumor suppressor genes, being potential treatments for cancers [61,62]. In the present work, except for H4K8 and H2BK20, the alteration in the acetylation level of other lysine residues, e.g., H4K16, H4K12, and K3K18, was not significantly changed in the presence of a low concentration of TSA. The comprehensive regulation of histone acetylation and the effectiveness of genotoxic platinum drugs require further studies. 

## 3. Materials and Methods

### 3.1. Chemicals and Materials

Deionized water produced by the Millipore system was used throughout the entire work. Cisplatin (DDP) and trichostatin A (TSA) were purchased from Innochem (Beijing, China). Ferrostatin-1 was purchased from Bidepharm (Shanghai, China). MS-grade acetonitrile and water were purchased from Fisher Chemical (Hampton, NH, USA). Primary antibodies that recognize cleaved caspase-3 (cat#9661), cleaved caspase-6 (cat#9761), phospho-CHK2 (Thr68) (cat#2197), and CD44 (cat#37259) were purchased from Cell Signaling Technology (Danvers, MA, USA). Antibodies recognizing ATM (cat#ab32420), CHK2 (cat#ab109413), CDK1 (cat#ab13327), phospho-Histone H2AX (Ser139) (cat#ab81299), phospho-ATM (Ser1981) (cat#ab81292), phospho-CDK1 (Tyr15) (cat#ab47549), and cisplatin-modified DNA (cat#ab103261) were purchased from Abcam (Cambridge, UK). Secondary antibodies including Goat Anti-Rabbit IgG H&L (HRP) (cat#ab6721), Donkey Anti-Rabbit IgG H&L (Alexa Fluor^®^488) (cat#ab150073), Goat Anti-Mouse IgG H&L (Cy5^®^) (cat#ab6563), and Goat Anti-Rat IgG H&L (Alexa Fluor^®^555) (cat#ab150158) were also provided by Abcam.

### 3.2. Cell Cultures

The human cancer cell lines, A549 cells, gastrointestinal stromal tumor (GIST) cells, HeLa ovarian cells, HepG2 hepatoma cells, and MCF-7 breast cancer cells (National Infrastructure of Cell Line Resource, Shanghai, China) were cultured in DMEM (Dulbecco’s Modifed Eagle’s Medium, Gibco, Waltham, MA, USA), which contained 10% fetal bovine serum (FBS, Gibco) and 1% penicillin–streptomycin (GE Heathcare Life Sciences, Piscataway, NJ, USA). Cisplatin-resistant A549 (A549/DDP) cells (National Infrastructure of Cell Line Resource, China) were cultured in RPMI-1640 (Roswell Park Memorial Institute-1640, Gibco, USA), which contained 10% fetal bovine serum and 1% penicillin–streptomycin. All cells were cultured in 5% CO_2_ in a 37 °C incubator.

### 3.3. CCK-8 Assay and Determination of IC50

A549 cells (5 × 10^3^) were seeded into each well of 96-well plates and cultured in DMEM for 24 h before the addition of the requested concentration of cisplatin with or without TSA. Then, the cells were cultured for 48 h before the viability assessment with cell counting kit-8 (CCK-8, MedChemExpress, Princeton, NJ, USA). Aliquot (10 µL) of CCK-8 reagent was added to each well described above for 3 h of further incubation. Then, the absorbance at 450 nm of each well was measured by a micro-plate reader (TECAN F50, Männedorf, Switzerland). Based on the absorbance and the dose of cisplatin, the inhibition curves on A549 cells were plotted using the Hill equation-based regression. IC_50_ was calculated from the regression equation, where the absorbance was 50% of the maximum reading.

### 3.4. Cell Apoptosis and Cell Cycle Assay

Cells were seeded at a density of 1 × 10^5^ cells/well in a six-well plate in triplicate and incubated in DMEM for 24 h. Then, cells were treated with TSA (1 µM) and/or DDP (8 µM). After 48 h incubation, cells were harvested, trypsinized, and washed with cold phosphate-buffered saline (PBS). Subsequently, for the apoptosis assay, cells were stained with FITC-Annexin V and propidium iodide (PI) (BD Bioscience Pharmingen, San Diego, CA, USA) for 5 min, the stained cells were immediately analyzed using the BD FACSCalibur™ Flow Cytometer (Becton, Dickinson and Company, San Diego, CA, USA), and the data were analyzed using FlowJo 10. For the cell cycle assay, the cells were fixed in cold ethanol overnight and stained with propidium iodide (PI) (Solarbio, Beijing, China) at 37 °C for 1 h, followed by washing three times with PBS. The stained cells were then analyzed using the BD FACSCalibur™ Flow Cytometer, and the data were analyzed using FlowJo 10.

### 3.5. Caspase Activity Assay

A549 cells were seeded in 6-well culture plates and incubated in DMEM supplemented with 10% FBS. After being treated as described above, proteins were extracted and measured by using the BCA Protein Assay kit. Caspase-3 activity was measured using the Caspase-3 Activity Assay kit (Beyotime Institute of Biotechnology, Nantong, China), in which cell extracts were mixed with Ac-DEVD-pNA substrate for 2 h at 37 °C in 96-well plates prior to the colorimetric measurement of p-nitroanilide product at 405 nm. Caspase-6 activity was measured using the Caspase-6 Activity Assay kit (Beyotime Institute of Biotechnology, China), in which cell extracts were mixed with Ac-VEID-pNA substrate for 2 h at 37 °C in 96-well plates prior to the colorimetric measurement of p-nitroanilide product at 405 nm.

### 3.6. Western Blotting

Cells treated with TSA and/or DDP were lysed with mammalian protein extraction reagent (Thermo Scientific, Waltham, MA, USA) containing a protease inhibitor cocktail (Beyotime, China). The lysate was quantified by the Enhanced BCA Protein Assay Kit (Beyotime). Aliquot (40 μg) of protein extract was boiled at 95 °C for 5 min with the gel-loading buffer (4 × LDS, Genscript, Nanjing, China). Subsequently, the protein samples were separated on SurePAGE 4–12% Bis-Tris gels (Genscript) and transferred to the PVDF membrane (Merck, Darmstadt, Germany) using the eBlot L1 Quick wet converter (GenScript). The blot was washed with TBS-0.1% Tween and blocked with 5% non-fat skim milk (Bio-Rad, Hercules, CA, USA) for 120 min at room temperature. The blot was then incubated with primary antibodies overnight at 4 °C. The next day, after washing three times with TBS-0.1% Tween, the blot was incubated with corresponding horseradish peroxidase-conjugated secondary antibodies. The enhanced chemiluminescence detection solution (ThermoFisher) was used to detect the immunoreactive bands.

### 3.7. Dot Blot Assay

Aliquot (1 μg) of DNA extracted from A549 cells treated under different conditions was boiled at 95 °C for 5 min; then, we used a pipette tip to spot the sample onto the PVDF membrane and dried the film at 60 °C for 30 min. The blots were blocked with 5% non-fat skim milk for 120 min at room temperature. Then, the blots were incubated with primary antibodies that recognize cisplatin-modified DNA overnight at 4 °C. The next day, after washing three times with TBS-0.1% Tween, the blots were incubated with horseradish peroxidase-conjugated goat anti-rat secondary antibodies. The enhanced chemiluminescence detection solution was used to detect the immunoreactive dots.

### 3.8. Immunofluorescence Imaging

After cells were treated with TSA (1 µM) and/or DDP (8 µM) for 48 h, the cells were treated with cold methanol–acetic acid fixative for 10 min, washed three times with PBS, permeabilized with 0.1% Triton-X 100/PBS for 30 min, and then blocked with 5% BSA/PBS solution at 37 °C for 1 h. Thereafter, the cells were incubated with primary antibody at 4 °C overnight, followed by washing three times with buffer (0.2% BSA/PBS). After that, the cells were incubated with a corresponding secondary antibody conjugated with a fluorophore at 37 °C for 1 h and then washed three times with buffer. Images of stained cells were recorded by a Confocal Laser Scanning Microscope (FLUOVIEW FV3000 series, Olympus, Tokyo, Japan).

### 3.9. Wound Healing Assay

Cells were seeded at a density of 1 × 10^5^ cells/well in a six-well plate in triplicate and incubated in DMEM for 24 h. Then, cells were treated with TSA (1 µM) and/or DDP (8 µM). After 48 h incubation. The cells adhering to each well were scratched with a pipette tip to generate a wound, and the wounded cells were washed three times with PBS, followed by the addition of fresh medium and further incubation of 36 h. Images of the cells were then taken by a high-resolution camera, and the wound area of cells on each well was measured to calculate the healing rate of wound cells by using Equation (1):

(1) $$Healing\;Rate=\frac{{WA}_{0}-{WA}_{36}}{{WA}_{0}}$$

where WA_0_ represents the wound area of cells on a well at 0 h; WA_36_ represents the wound area of the cells at 36 h. 

### 3.10. ROS and Lipid Peroxidation Detection

Cells were treated with 8 μM of DDP in the absence and presence of 1 μM of TSA for 48 h; then, cells were washed with PBS. The fluorescent probe DCFH-DA of the Reactive Oxygen Species Assay Kit (Solarbio) or liperfluo of lipid peroxidation (DOJINDO, Kumamoto, Japan) was used in a serum-free medium with a 1:1000 dilution. After incubation at 37 °C for 20 min, cells were washed with serum-free medium 3 times prior to LSCM imaging.

### 3.11. Inductively Coupled Plasma Mass Spectrometry (ICP-MS)

After treatment with TSA (1 µM) and/or DDP (8 µM) for 48 h, cells were individually collected for each group, counted by the LUNA-II™ Automated Cell Counter (Logos Biosystems, Solana Beach, CA, USA), and digested by 20% HNO_3_ at 200 °C. Finally, the residue was re-dissolved in 1% HNO_3_, and ICP-MS (Agilent 7700, Santa Clara, CA, USA) was used to determine the concentration of Pt in the cells. In order to measure the concentration of DNA-bound platinum, nuclear DNA in each group of cells was extracted using the Nuclear Extraction Kit (BestBio, Shanghai, China) and Genomic DNA Mini Preparation Kit with Spin Column (Beyotime). The concentration of DNA in each extract was detected by NanoDrop (ThermoFisher Scientific) before the concentration of Pt was determined by ICP-MS. The measurements for Pt concentration in whole cells and bound with DNA were repeated three times independently, and the values were represented as the amount of Pt (ng) per 10^6^ cells or Pt (ng) per mg DNA.

### 3.12. Time of Flight Secondary Ion Mass Spectrometry (ToF-SIMS) Imaging

For ToF-SIMS imaging, A549 cells were seeded on silicon wafers, cultivated to an 80–90% coverage, and divided into four groups: control group (cells cultivated without DDP or TSA), TSA group (cells treated with 1 μM of TSA for 48 h), DDP group (cells treated with 8 μM of DDP for 48 h), and DDP + TSA group (cells treated with 8 μM of DDP and 1 μM of TSA for 48 h). Each group of cells was individually fixed with pure, pre-cooled methanol for 15 min at −20 °C and washed three times with PBS, followed by washing three times with ammonium acetate (150 mM, pH 7.4) and being quickly frozen by liquid N_2_. The frozen cells were transferred immediately into a lyophilizer (LGJ-12, Beijing Songyuanhuaxing Technology Develop Co., Ltd., Beijing, China) at 193 to 208 K for freeze-drying overnight.

ToF-SIMS imaging was carried out with a ToF-SIMS 5 instrument (ION-ToF GmbH, Münster, Germany) equipped with a 30 keV Bismuth liquid metal primary ion source by following a similar program reported in our previous works [5,26]. In brief, high-lateral-resolution (200–300 nm) images of cells were recorded using a Bi_3_^+^ primary ion gun, and signals were collected with 256 × 256 pixels in negative mode; the scanning area was 250 μm × 250 μm. Each sample was scanned 500 times to obtain high-quality images. The images of various ions were collected and plotted with Surface Lab software (version 6.8 ION-ToF GmbH). The mass-to-charge ratio (*m*/*z*) was calibrated using the signals of C^−^, CH^−^, C_2_^−^, and C_2_H^−^. The image of Pt was constructed by the sum of signals of [^194^PtCN]^−^, [^195^PtCN]^−^, and [^196^PtCN]^−^ ions, and the image of total ions and PO_3_^−^ was used to profile the shape of the cell and nucleus. Shift correction was applied by the software for all the images. A region of interest (ROI) was created for 20 cells in each sample, and the Pt signal of each cell (or ROI) was normalized to the signal intensity of PO_3_^−^ for further statistics analysis.

### 3.13. Preparation of Samples for Quantitative Proteomics Analysis

Three groups of A549 cells were cultivated with 8 μM of DDP alone (designated as the DDP group), 1 μM of TSA alone (designated as the TSA group), or 8 μM of DDP and 1 μM of TSA (designated as the DDP + TSA group) for 48 h. Each group had three independent replicates. The cells were then individually harvested and lysed with a mammalian protein extraction reagent (Thermo Fisher Scientific, USA) containing a protease inhibitor cocktail (Beyotime, China) and 8 M urea (Solarbio, China). The concentration of all proteins in each lysate was quantified by the Enhanced BCA Protein Assay Kit (Beyotime).

The tryptic digestion of extract proteins, tandem-mass-tag (TMT) labeling, and pre-fractionalization of tryptic peptides were performed by following the procedure described in our previous works [5,26]. Exactly 500 μg of extracted proteins in lysis buffer from each group was transferred to a 1.5 mL low-protein-binding microcentrifuge tube (Thermo Fisher Scientific), and TCEP (Thermo Fisher Scientific) was added at a final concentration of 5 μM. The resulting mixture was reacted at 37 °C for 1 h. Then, IAA (Tokyo Chemical Industry Shanghai, China) was added at a final concentration of 10 μM to alkylate the cysteine residues by incubation at 25 °C for 45 min in the dark. After diluting 3 times with 50 mM Tris-HCl (pH 8.0), 10 μL of 1 μg/μL trypsin was added to digest proteins at 25 °C for 14 h. Thereafter, the peptides were desalted in a C18 cartridge (Waters Sep-Pak, Part No. WAT023590, Framingham, MA, USA). The C18 column was first activated by 1 mL of acetonitrile (ACN) and 1 mL of 50% (vol/vol) ACN/H_2_O with 0.1% (vol/vol) formic acid (FA) successively, followed by equilibration with 3 mL of 0.1% (vol/vol) TFA (trifluoacetic acid) in H_2_O. Then, tryptic peptides derived from each sample were loaded to the C18 column, and desalting was achieved by washing the column with 3 mL of 0.1% (vol/vol) TFA and 1 mL of 1% (vol/vol) FA. The peptide residues were sequentially eluted by 1 mL of 50% (vol/vol) ACN and 1 mL of 80% (vol/vol) ACN/H_2_O, and the eluents were merged, followed by drying in a vacuum centrifuge (CentriVap, Thermo Fisher Scientific).

Exactly 100 μg of peptide residues of each sample was re-dissolved in 50 mM HEPES (pH 8.5) to a final concentration of 1 μg/μL and labeled with tandem-mass-tag (TMT) labeling reagent (Thermo Fisher Scientific). The samples of the DDP group were labeled as 130N, 130C, and 131 and the DDP + TSA group were labeled as 128C, 129N, and 129C. The labeling reaction was carried out by the incubation of peptides with TMT reagents at room temperature for 1 h and quenched by adding 8 μL of 5% hydroxylamine. The labeled peptides derived from the DDP group and DDP + TSA group were then equivalently mixed and dried as described above.

The labeled peptide mixture was re-dissolved in 100 μL 2% (vol/vol) ACN/H_2_O containing 4.5 mM ammonium formate (pH 10) for pre-fractionation in basic reverse-phase chromatography. Aliquot (97 μL) of the peptide mixture was loaded to HPLC (Agilent Technologies 1260 infinity, USA) with an Agilent ZORBAX 300 Extend-C18 column. The mobile phase A was 4.5 mM ammonium formate in 2% (vol/vol) ACN/H_2_O (pH 10) and phase B was 4.5 mM ammonium formate in 90% (vol/vol) ACN/H_2_O (pH 10). The gradient started with 0% B until 7 min and continuously increased to 16% B at 13 min, 40% B at 73 min, 44% B at 77 min, and 60% B at 82 min, which was kept until 96 min and then increased to 90% B at 100 min. The flow rate was 1 mL/min. T The fractions were collected chronologically into twelve tubes from 3–96 min on the basis of crossfading fractionation [63]. The pooled fractions were spun to dry them and re-dissolved in H_2_O containing 0.1% FA to a 500 μg/μL concentration of peptides for mass spectrometry analysis.

### 3.14. Quantitative Proteomics Analysis

Mass spectrometry (MS)-based quantitative proteomics analysis was performed on an Orbitrap Fusion Lumos mass spectrometer coupled with an EASY-nLC 1200 nanoUPLC system equipped with an Acclaim™ PepMap™ 100 pre-column (20 mm × 75 μm, 3 μm) and an Acclaim™ PepMap™ RSLC C18 analytical column (150 mm × 75 μm, 2 μm). The UPLC mobile phase A was water containing 0.1% FA and phase B was 80% (vol/vol) methanol/water containing 0.1% FA. The UPLC gradient started with 2% B and increased to 7% at 7 min, 20% at 69 min, 35% at 90 min, and sharply to 95% within 5 min; this remained for 4 min and then finally decreased to 2% within 8 min and remained for 3 min. Aliquot (1 μL) of each HPLC fraction described above was injected into UPLC and the elution from the analytical column was directly infused into the mass spectrometer for MS/MS analysis. 

The voltage of electrospray ionization (ESI) was set as 2200 V and the ion transfer tube temperature was 320 °C. For primary mass spectrometry analysis, an Orbitrap detector was operated with a mass resolution of 120,000 and a scanning range from 350 to 1800 *m*/*z.* The Orbitrap was also used as the detector of MS/MS with a mass resolution of 15,000 and of MS^3^ with a resolution of 50,000 and a scanning range of 100–200 *m*/*z*. The HCD fragmentation cell ran with a collision energy of 23% for the second mass spectrometry (MS/MS) analysis and with a collision energy of 60% for the third mass spectrometry (MS^3^) analysis.

Raw MS/MS data were searched in the Proteome Discoverer (Thermo Scientific, version 2.4) database for peptide and protein identification. The Sequest HT search engine was used for peptide–spectrum matching (PSM). The dynamic modifications were oxidation at methionine; methylation at lysine, glutamine, and arginine; acetylation at lysine and serine; phosphorylation at serine, threonine, and tyrosine; and TMT labeling at lysine. The static modifications were carbamidomethylation at cysteine and TMT labeling at the N-terminus of peptides. The quantitative results were normalized based on the total peptide amount in each group. Only proteins identified with a false discovery rate (FDR) < 0.01, *p*-value < 0.05, and abundance ratio < 0.833 or > 1.200 were identified as differentially expressed proteins (DEPs) between two groups of cells and pooled for further bioinformatics analyses.

## 4. Conclusions

Given that histones play an important role in the assembling and structure of nucleosomes, the acetylation of lysine residues in histones was shown to significantly regulate the cytotoxicity of genotoxic anticancer drugs, for instance, cisplatin. In the present work, by using trichostatin A (TSA) as a histone deacetylase inhibitor, we applied mass spectrometry-based quantitative proteomics analysis to decipher the underlying mechanism by which histone acetylation regulates the cytotoxicity of cisplatin, combined with the use of molecular biological methods and bioinformatics analysis techniques. We revealed that a low concentration of TSA promoted cisplatin-induced apoptosis, evidenced by the activation of caspase-3 and -6, which subsequently cleaved PARP1 and increased the degradation of lamin A&C. The TSA-induced promotion of cisplatin activity activated the ATM-CHK2 pathway, causing more arrest of A549 cells at the G2/M phase. The significant increase in platinum-induced DNA lesions was due to TSA-induced alternation in nucleosomal DNA structures, which was evidenced by the elevation of the DNA damage marker γH2AX, and this is the major contributor to the promotion of cisplatin cytotoxicity. 

Our global quantitative proteomics data showed that in the presence of TSA, cisplatin activated INF signaling to upregulate STAT1 and SAMHD1 to promote cisplatin sensitivity and downregulated ICAM1 and CD44 to reduce cell migration, synergistically promoting cisplatin cytotoxicity. Furthermore, in the presence of TSA, cisplatin downregulated TFAM and SLC3A2 to enhance cisplatin-induced ferroptosis, evidenced by the increase in the levels of ROS and lipid peroxidation. More importantly, our posttranslational modification data demonstrated that acetylation at H4K8 played a dominant role in promoting cisplatin cytotoxicity. These findings provide novel insights into better understanding the principle of combining chemotherapy for the treatment of cancers. We are aware that in vivo studies and more in vitro studies, such as of CHIP-seq and site-directed mutation on H4K8, could further validate the precise correlation of the acetylation of histones and the cytotoxicity of platinum drugs, which will be the topic of our future work. 

## Figures and Tables

**Table 1:** The half-maximal inhibition concentration (IC_50_) of cisplatin for various human cancer cell lines in the absence (−) or presence (+) of 1 μM of TSA.

Cell Line	IC_50_ (μM) of Cisplatin		SI ^1^
	−TSA	+TSA	
A549	7.8 ± 0.6	2.3 ± 0.4	3.4
A549/DDP	46.7 ± 2.5	35.2 ± 2.2	1.3
GIST	11.8 ± 0.9	8.1 ± 0.8	1.5
HeLa	10.2 ± 0.8	7.2 ± 1.0	1.4
HepG2	17.3 ± 2.2	13.1 ± 1.2	1.3
MCF7	14.3 ± 2.9	11.1 ± 0.8	1.3

^1^ SI represents sensitivity index, equal to IC_50_ (−TSA)/IC_50_ (+TSA).

## Data Availability

All raw MS data are available via ProteomeXchange with identifiers PXD052203 and PXD052305.

## Supplementary Materials

The following supporting information can be downloaded at https://www.mdpi.com/article/10.3390/molecules29112623/s1: Figure S1: IC50 curves of TSA to A549 cell in 48h; Figure S2: Effects of TSA and DDP on cell cycle of A549 cells treated with 8 μM DDP in the absence or the presence of 1 μM TSA for 48 h; Figure S3: Would healing assay. Figure S4: IPA enriched canonical signaling pathways with which the differentially expressed proteins (DEPs) with |FC| > 1.2 identified in A549 cells treated with TSA (TSA) and DDP (b) only, compared with the proteins expressed in the control group of A549 cells. Figure S5: MS/MS spectrum of the peptide used to identify H4K8 acetylation. Table S1: MS/MS raw data for protein identification and quantification in A549 cells treated with DDP in the absence or the absence of TSA with *p* < 0.05; Table S2: Differentially expressed proteins (DEPs) with |FC| ≥ 1.2 and *p*-value < 0.05 identified in A549 cells treated with DDP (8 μM) and TSA (1 μM), in comparison with those in A549 cells treat with DDP (8 μM) alone; Table S3: MS/MS raw data for protein identification and quantification in A549 cells treated with TSA with *p* < 0.05. Table S4: Differentially expressed proteins (DEPs) with |FC| ≥ 1.2 and *p*-value < 0.05 identified in A549 cells treated with TSA (1.0 μM), in comparison with those in control group of A549 cells; Table S5: MS/MS raw data for protein identification and quantification in A549 cells treated with DDP with *p* < 0.05; Table S6: Differentially expressed proteins (DEPs) with |FC| ≥ 1.2 and *p*-value < 0.05 identified in A549 cells treated with DDP (8.0 μM), in comparison with those in control group of A549 cells; Table S7: MS/MS raw data for identification and quantification of histone acetylation level in A549 cells treated with DDP in the absence or the absence of TSA; Table S8: Histone acetylation level identified and quantified in A549 cells treated with DDP (8 μM) and TSA (1 μM), in comparison with those in A549 cells treat with DDP (8 μM) alone.
