# Supplementary material for: Trichostatin A Promotes Cytotoxicity of Cisplatin, as Evidenced by Enhanced Apoptosis/Cell Death Markers
Source: Molecules. 2024 Jun 3;29(11):2623. doi: 10.3390/molecules29112623 (PMC11173726; doi:10.3390/molecules29112623)
Supplement: Supplementary file 1 [file molecules-29-02623-s001.zip › supporting information.pdf]

Supporting Information

Trichostatin A Promotes Cytotoxicity of Cisplatin by Enhancing Apoptosis/Ferroptosis and Reducing Cell Migration

Yang Zhou,[1,2] Qun Luo,[1,2] Fangang Zeng,[3] Xingkai Liu,[1] Juanjuan Han,[1,4] Liangzhen Gu,[1,2] Xiao Tian,[1,5] Yanyan Zhang,[1] Yao Zhao,[1,2,*] and Fuyi Wang[1,2,4,5,*]

[1] *Beijing National Laboratory for Molecular Sciences; CAS Research/Education Center for Excellence in Molecular Sciences; National Centre for Mass Spectrometry in Beijing; CAS Key Laboratory of Analytical Chemistry for Living Biosystems, Institute of Chemistry, Chinese Academy of Sciences, Beijing, 100190, P. R. China*

[2] *University of Chinese Academy of Sciences, Beijing, 100049, P.R. China*

[3] *School of Environment of Natural Resources, Remin University of China, Beijing, 100875, P.R. China.*

[4] *National Centre for Mass Spectrometry in Beijing, Beijing, 100190, P. R. China*

[5] *College of Traditional Chinese Medicine, Shandong University of Traditional Chinese Medicine, Jinan, 250355, P.R. China*

* Corresponding authors: E-mail: yaozhao@iccas.ac.cn; fuyi.wang@iccas.ac.cn

Figure S1 – Figure S5

Table S1 – Table S8

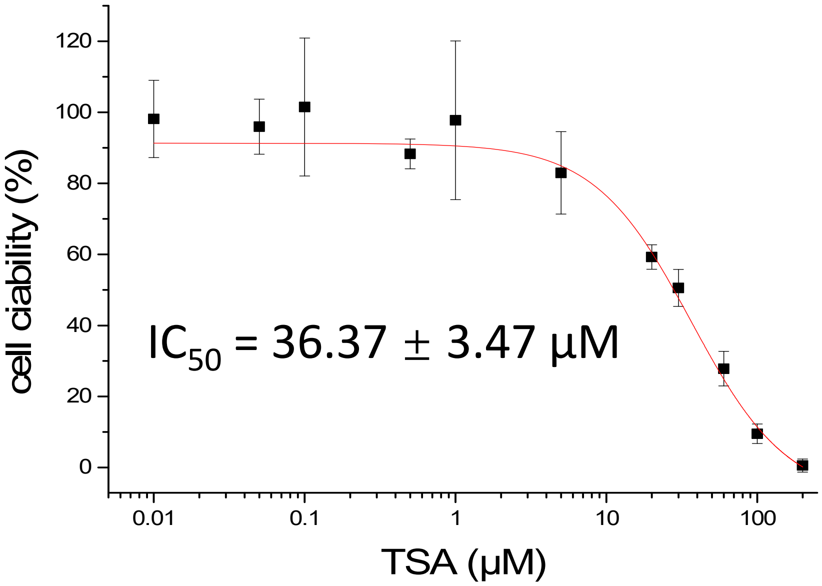

Figure S1. IC_{50} curves of TSA to A549 cell in 48h.

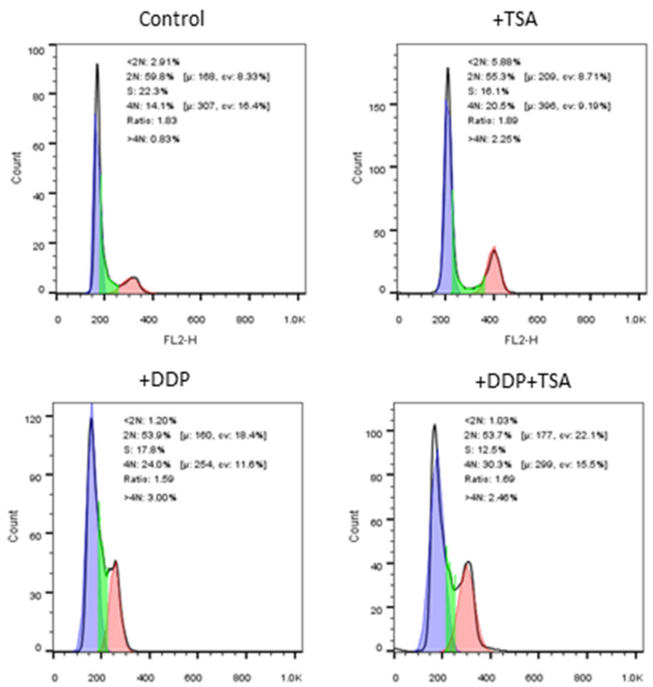

Figure S2. Effects of TSA and DDP on cell cycle of A549 cells treated with 8 μM DDP in the absence or the presence of 1 μM TSA for 48 h.

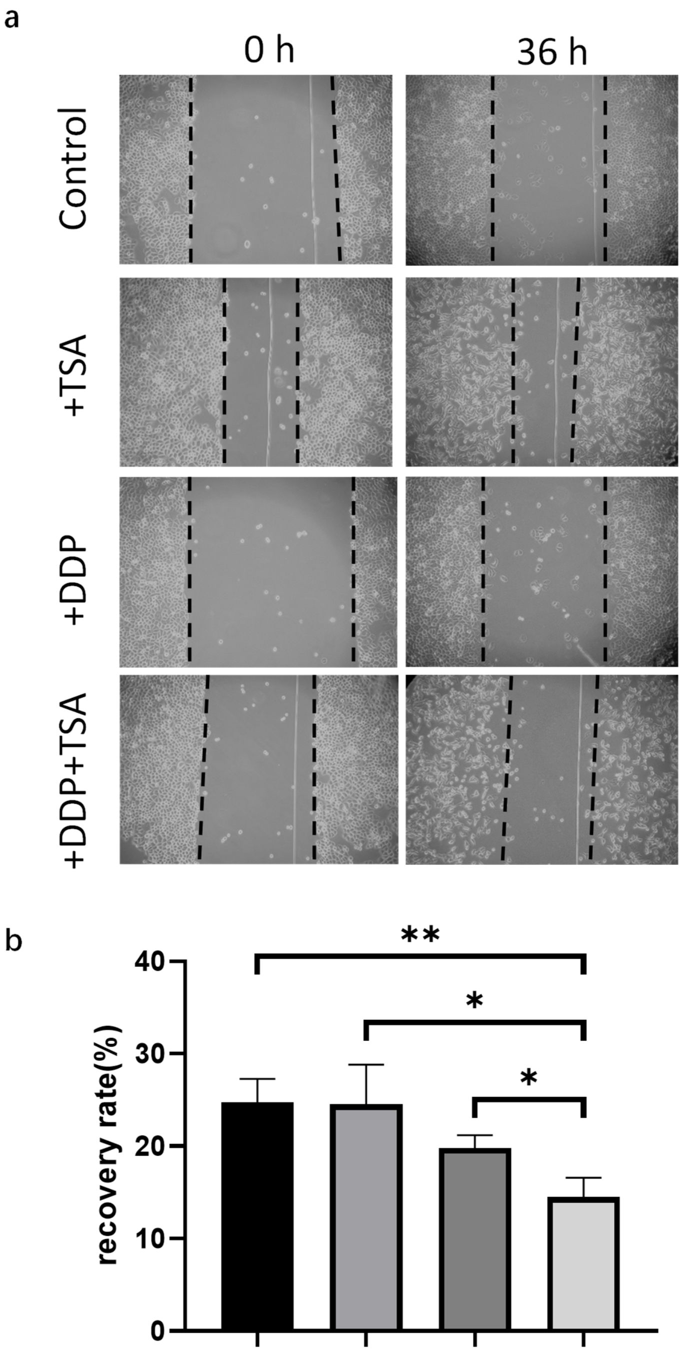

Figure S3. Would healing assay. (a) Bright field optical images showing the wound healing of A249 cells treated under different conditions. (b) Histogram of wound healing rate of A549 cells treated under different conditions (n = 3). The data are presented as means ± SD; *$p < 0.05$, **$p < 0.01$ (Student's t test).

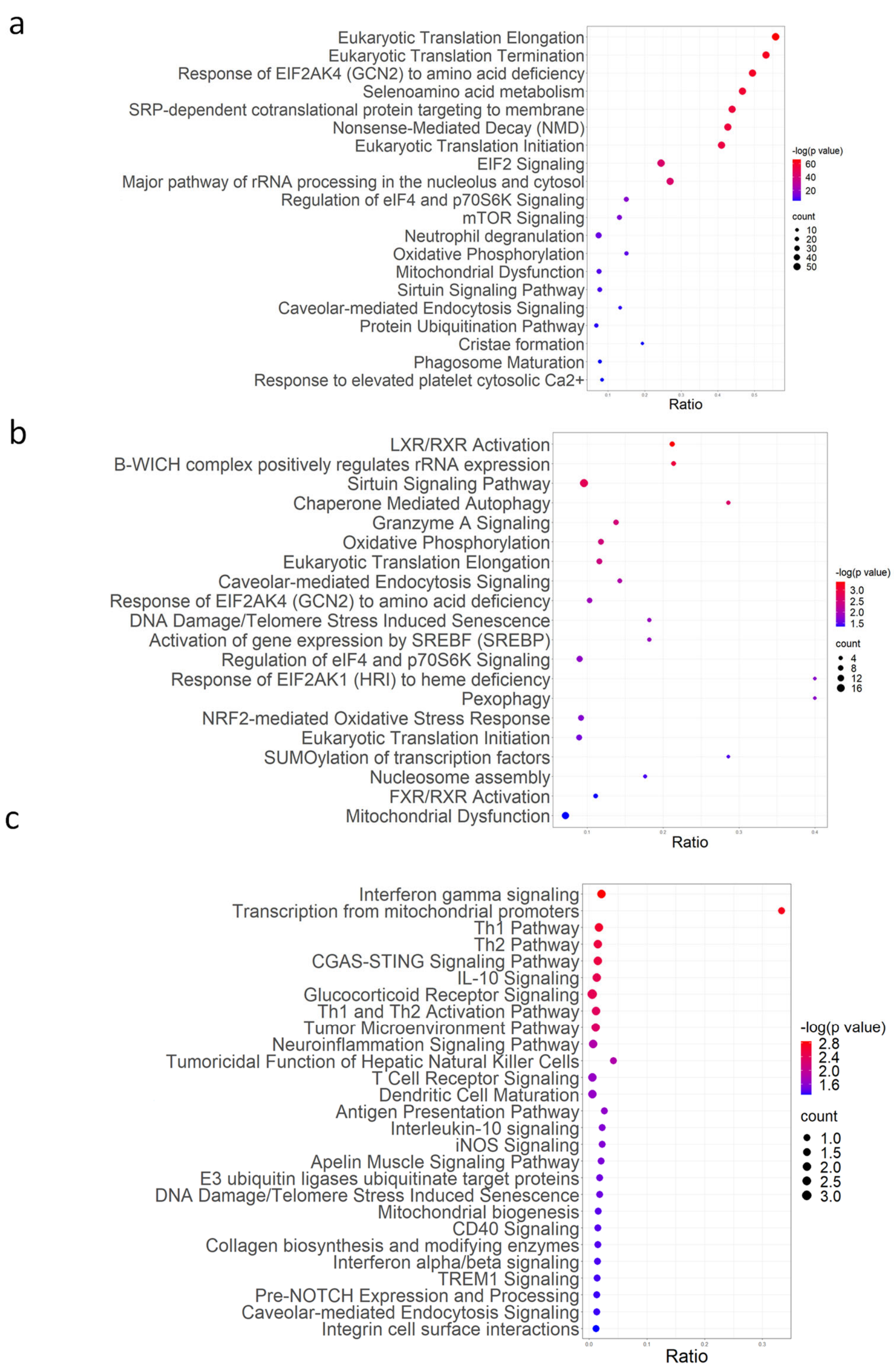

Figure S4. IPA enriched canonical signaling pathways with which the differentially expressed proteins (DEPs) with |FC| > 1.2 identified in A549 cells treated with TSA (TSA) and DDP (b) only, compared with the proteins expressed in the control group of A549 cells.

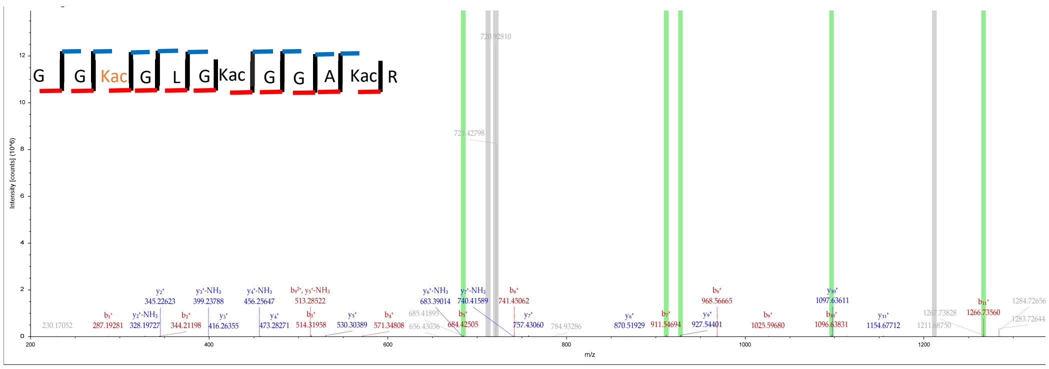

Figure S5. MS/MS spectrum of the peptide used to identify H4K8 acetylation.

Table S1. MS/MS raw data for protein identification and quantification in A549 cells treated with DDP in the absence or the absence of TSA with $p < 0.05$.

See this table on a separated Excel file named Supporting information Table S1

Table S2. Differentially expressed proteins (DEPs) with $|FC| \geq 1.2$ and p-value <0.05 identified in A549 cells treated with DDP (8 μM) and TSA (1 μM), in comparison with those in A549 cells treat with DDP (8 μM) alone.

Accession	Gene Symbol	Description	Fold Change
O94811	TPPP	Tubulin polymerization-promoting protein	2.23
P36952-1	SERPINB5	Serpin B5	1.82
Q6ZMI0-1	PPP1R21	Protein phosphatase 1 regulatory subunit 21	1.76
Q9Y2W6	TDRKH	tudor and KH domain-containing protein	1.74
P50336	PPOX	protoporphyrinogen oxidase	1.59
P08243-1	ASNS	Asparagine synthetase [glutamine-hydrolyzing]	1.57
Q9Y2D0	CA5B	Carbonic anhydrase 5B, mitochondrial	1.54
O95671-1	ASMTL	N-acetylserotonin O-methyltransferase-like protein	1.52
O43301	HSPA12A	Heat shock 70 kDa protein 12A	1.51
P22033	MMUT	Methylmalonyl-CoA mutase, mitochondrial	1.48
Q92947-1	GCDH	Glutaryl-CoA dehydrogenase, mitochondrial	1.46
P15374	UCHL3	Ubiquitin carboxyl-terminal hydrolase isozyme L3	1.46
Q14657	LAGE3	EKC/KEOPS complex subunit LAGE3	1.44
Q02252	ALDH6A1	Methylmalonate-semialdehyde dehydrogenase [acylating], mitochondrial	1.41
Q04695	KRT17	Keratin, type I cytoskeletal 17	1.41
Q9UBQ6	EXTL2	Exostosin-like 2	1.41
P01350	GAST	Gastrin	1.40
Q9Y3Z3	SAMHD1	deoxynucleoside triphosphate triphosphohydrolase SAMHD1	1.39
Q15404-1	RSU1	Ras suppressor protein 1	1.37

P57105	SYNJ2BP	Synaptojanin-2-binding protein	1.36
Q9H223	EHD4	EH domain-containing protein 4	1.34
Q9H7C9-1	AAMDC	Mth938 domain-containing protein	1.33
P48735	IDH2	Isocitrate dehydrogenase [NADP], mitochondrial	1.33
Q6UW78	UQCC3	Ubiquinol-cytochrome-c reductase complex assembly factor 3	1.33
Q14240-1	Eif4a2; EIF4A2; LOC101352730; LOC111152235	eukaryotic initiation factor 4A-II	1.32
Q13509	TUBB3	tubulin beta-3 chain	1.31
Q3SXM5	HSDL1	Inactive hydroxysteroid dehydrogenase-like protein 1	1.31
Q9NQG1	MANBAL	Protein MANBAL	1.30
Q9Y624	F11R	Junctional adhesion molecule A	1.30
P51648	ALDH3A2	Fatty aldehyde dehydrogenase	1.29
P11413-1	G6PD	Glucose-6-phosphate 1-dehydrogenase	1.28
P55795	HNRNPH2	heterogeneous nuclear ribonucleoprotein H2	1.27
O43813	LANCL1	LanC-like protein 1	1.26
P83436	COG7	Conserved oligomeric Golgi complex subunit 7	1.26
P78540	ARG2	arginase-2, mitochondrial	1.26
Q9UKM9-1	RALY	RNA-binding protein Raly	1.25
P42224-1	STAT1	Signal transducer and activator of transcription 1-alpha/beta	1.24
P13804-1	ETFA	Electron transfer flavoprotein subunit alpha, mitochondrial	1.24
P49748-1	ACADVL	Very long-chain specific acyl-CoA dehydrogenase, mitochondrial	1.24
O76027	ANXA9	Annexin A9	1.24
O15400	STX7	Syntaxin-7	1.22
O15479	MAGEB2	Melanoma-associated antigen B2	1.22

Q8N4Q1	CHCHD4	Mitochondrial intermembrane space import and assembly protein 40	1.22
Q96RP9	GFM1	Elongation factor G, mitochondrial	1.21
O43143	DHX15	Pre-mRNA-splicing factor ATP-dependent RNA helicase DHX15	1.21
Q8NHP6-1	MOSPD2	motile sperm domain-containing protein 2	1.20
Q9NPJ3-1	ACOT13	Acyl-coenzyme A thioesterase 13	1.20
Q8IV38	ANKMY2	Ankyrin repeat and MYND domain-containing protein 2	1.20
P15291	B4GALT1	Beta-1,4-galactosyltransferase 1	-1.20
P08195	SLC3A2	4F2 cell-surface antigen heavy chain	-1.20
Q9BRG1	VPS25	Vacuolar protein-sorting-associated protein 25	-1.20
O75691	UTP20	Small subunit processome component 20 homolog	-1.21
Q9NR30-1	DDX21	Nucleolar RNA helicase 2	-1.22
Q96II8-1	LRCH3	Leucine-rich repeat and calponin homology domain-containing protein 3	-1.22
P38432	COIL	coilin	-1.24
P30520	ADSS2	adenylosuccinate synthetase isozyme 2	-1.24
O75781	PALM	Paralemmin-1	-1.25
O94913	PCF11	Pre-mRNA cleavage complex 2 protein Pcf11	-1.25
Q9H488-1	POFUT1	GDP-fucose protein O-fucosyltransferase 1	-1.26
Q8TED1	GPX8	Probable glutathione peroxidase 8	-1.26
Q9NRK6	ABCB10	ATP-binding cassette sub-family B member 10, mitochondrial	-1.26
P01891	HLA-A	HLA class I histocompatibility antigen, A-68 alpha chain	-1.27
Q9UG63	ABCF2	ATP-binding cassette sub-family F member 2	-1.29

P18754-1	RCC1	Regulator of chromosome condensation	-1.29
Q8N129	CNPY4	Protein canopy homolog 4	-1.30
P62805	H4C1; H4C11; H4C12; H4C13; H4C14; H4C15; H4C16; H4C2; H4C3; H4C4; H4C5; H4C6; H4C8; H4C9	histone H4	-1.31
O75665	OFD1	Oral-facial-digital syndrome 1 protein	-1.31
Q8NBL1	POGLUT1	Protein O-glucosyltransferase 1	-1.31
Q9UBV8	PEF1	Peflin	-1.32
Q00059	TFAM	Transcription factor A, mitochondrial	-1.32
O95302	FKBP9	Peptidyl-prolyl cis-trans isomerase FKBP9	-1.33
Q14696	MESD	LRP chaperone MESD	-1.33
P17096-1	HMGA1; LOC111152215	High mobility group protein HMG-I/HMG-Y	-1.33
Q5SSJ5-1	HP1BP3	Heterochromatin protein 1-binding protein 3	-1.34
Q9BZE2	PUS3	tRNA pseudouridine(38/39) synthase	-1.34
P16070	CD44	CD44 antigen	-1.35
P05204	HMGN2	Non-histone chromosomal protein HMG-17	-1.35
Q8WUH1	CHURC1; FNTB	Protein Churchill	-1.36
P02786	TFRC	Transferrin receptor protein 1	-1.39
O75367-1	MACROH2A1	Core histone macro-H2A.1	-1.41
O75955	FLOT1	Flotillin-1	-1.41
Q15293	RCN1	Reticulocalbin-1	-1.42
P05362	ICAM1	Intercellular adhesion molecule 1	-1.43
Q9Y2B0	CNPY2	Protein canopy homolog 2	-1.46
O00214-1	LGALS8	Galectin-8	-1.48
Q9BU89	DOHH	Deoxyhypusine hydroxylase	-1.50
Q7Z4H8	POGLUT3	KDEL motif-containing protein 2	-1.50
P26885	FKBP2	Peptidyl-prolyl cis-trans isomerase FKBP2	-1.51

O14657	TOR1B	Torsin-1B	-1.51
Q5VT79	ANXA8L1	annexin A8-like protein 1	-1.52
P17813	ENG	Endoglin	-1.53
Q9BX59	TAPBPL	Tapasin-related protein	-1.60
Q58FG1	HSP90AA4P	Putative heat shock protein HSP 90-alpha A4	-1.72
P50454	SERPINH1	Serpin H1	-1.79
P15923	TCF3	Transcription factor E2-alpha	-3.21

Table S3. MS/MS raw data for protein identification and quantification in A549 cells treated with TSA with $p < 0.05$.

See this table on a separated Excel file named Supporting information Table S3

Table S4. Differentially expressed proteins (DEPs) with |FC| ≥1.2 and p-value <0.05 identified in A549 cells treated with TSA (1.0 μM), in comparison with those in control group of A549 cells.

Accession	Gene Symbol	Description	Fold Change
P02647	APOA1	Apolipoprotein A-I	3.74
Q8IUE6	H2AC21	Histone H2A type 2-B	3.12
P68871	HBB	Hemoglobin subunit beta	2.87
P05204	HMGN2	Non-histone chromosomal protein HMG-17	2.86
P62805	H4C1; H4C2; H4C3; H4C4; H4C5; H4C6; H4C8; H4C9; H4C11; H4C12; H4C13; H4C14; H4C15; H4C16	histone H4	2.86
Q99878	H2AC14	Histone H2A type 1-J	2.69
Q00059	TFAM	Transcription factor A, mitochondrial	2.69
P02792	FTL	Ferritin light chain	2.66
P17096-1	HMGA1	High mobility group protein HMG-I/HMG-Y	2.59
Q71UI9-1	H2AZ2	Histone H2A.V	2.07
P02749	APOH	Beta-2-glycoprotein 1	1.94
P09525	ANXA4	annexin A4	1.88
P14314	PRKCSH	Glucosidase 2 subunit beta	1.86
P04183	TK1	Thymidine kinase, cytosolic	1.81
Q9GZQ8	MAP1LC3B	Microtubule-associated proteins 1A/1B light chain 3B	1.77

P30101	PDIA3	Protein disulfide-isomerase A3	1.75
P49006	MARCKSL1	MARCKS-related protein	1.73
P07099	EPHX1	epoxide hydrolase 1	1.73
P30040-1	ERP29	Endoplasmic reticulum resident protein 29	1.72
P08133-1	ANXA6	annexin A6	1.71
Q14697-1	GANAB	Neutral alpha-glucosidase AB	1.69
Q9HAW4	CLSPN	Claspin	1.69
Q9BT09	CNPY3	Protein canopy homolog 3	1.68
Q15293	RCN1	Reticulocalbin-1	1.67
Q969H8	MYDGF	Myeloid-derived growth factor	1.66
Q13162	PRDX4	Peroxiredoxin-4	1.66
Q15084-1	PDIA6	Protein disulfide-isomerase A6	1.65
Q96HE7	ERO1A	ERO1-like protein alpha	1.65
Q8NBS9-1	TXNDC5	Thioredoxin domain-containing protein 5	1.64
P61769	B2M	Beta-2-microglobulin	1.64
P11021	HSPA5	78 kDa glucose-regulated protein	1.63
P07237	P4HB	Protein disulfide-isomerase	1.62
O95881	TXNDC12	Thioredoxin domain-containing protein 12	1.62
P00367	GLUD1	Glutamate dehydrogenase 1, mitochondrial	1.61
P08962	CD63	CD63 antigen	1.61
P20073-1	ANXA7	Annexin A7	1.60
P02768-1	ALB	Serum albumin	1.60
O43852-4	CALU	Isoform 4 of Calumenin	1.60
Q99541	PLIN2	perilipin-2	1.60
Q16763	UBE2S	ubiquitin-conjugating enzyme E2 S	1.59
P04040	CAT	catalase	1.59
P04179	SOD2	Superoxide dismutase [Mn], mitochondrial	1.59
Q9UMX5	NENF	Neudesin	1.58
P09874	PARP1	Poly [ADP-ribose] polymerase 1	1.58
P07355	ANXA2	Annexin A2	1.58
Q99584	S100A13	Protein S100-A13	1.57
Q9NWM8	FKBP14	Peptidyl-prolyl cis-trans isomerase FKBP14	1.57
O43852	CALU	Calumenin	1.56
O75718	CRTAP	Cartilage-associated protein	1.56
Q9H488-1	POFUT1	GDP-fucose protein O-fucosyltransferase 1	1.55
P11388-1	TOP2A	DNA topoisomerase 2-alpha	1.54

P05362	ICAM1	Intercellular adhesion molecule 1	1.54
P25815	S100P	Protein S100-P	1.53
Q13885	TUBB2A	Tubulin beta-2A chain	1.53
P60903	S100A10	Protein S100-A10	1.53
P21580	TNFAIP3	Tumor necrosis factor alpha-induced protein 3	1.51
Q13530	SERINC3	Serine incorporator 3	1.50
P27797	CALR	Calreticulin	1.50
Q8IV08	PLD3	Phospholipase D3	1.49
Q9H7S9	ZNF703	Zinc finger protein 703	1.49
P30533	LRPAP1	alpha-2-macroglobulin receptor-associated protein	1.48
O75340	PDCD6	programmed cell death protein 6	1.48
Q96AY3	FKBP10	Peptidyl-prolyl cis-trans isomerase FKBP10	1.47
P10809	HSPD1	60 kDa heat shock protein, mitochondrial	1.47
Q8NEM2	SHCBP1	SHC SH2 domain-binding protein 1	1.47
P13667	PDIA4	Protein disulfide-isomerase A4	1.47
P08758	ANXA5	annexin A5	1.47
O00264	PGRMC1	Membrane-associated progesterone receptor component 1	1.46
Q9UBQ6	EXTL2	Exostosin-like 2	1.46
P30626-1	SRI	Sorcin	1.46
Q9Y2B0	CNPY2	Protein canopy homolog 2	1.46
P14625	HSP90B1	Endoplasmin	1.45
P06280	GLA	alpha-galactosidase A	1.45
Q8NI22	MCFD2	Multiple coagulation factor deficiency protein 2	1.44
P61916	NPC2	Epididymal secretory protein E1	1.44
O95302	FKBP9	Peptidyl-prolyl cis-trans isomerase FKBP9	1.44
Q9NP58	ABCB6	ATP-binding cassette sub-family B member 6, mitochondrial	1.44
Q16134	ETFDH	Electron transfer flavoprotein-ubiquinone oxidoreductase, mitochondrial	1.44
Q7Z4H8	POGLUT3	KDEL motif-containing protein 2	1.44
P13674-1	P4HA1	prolyl 4-hydroxylase subunit alpha-1	1.44

Q99643	SDHC	Succinate dehydrogenase cytochrome b560 subunit, mitochondrial	1.44
P06703	S100A6	protein S100-A6	1.44
Q8NI60-1	COQ8A	Atypical kinase COQ8A, mitochondrial	1.43
O43399	TPD52L2	Tumor protein D54	1.43
Q96EA4	SPDL1	protein Spindly	1.43
Q96D15	RCN3	Reticulocalbin-3	1.43
Q9Y277	VDAC3	Voltage-dependent anion-selective channel protein 3	1.43
L0R6Q1	SLC35A4	SLC35A4 upstream open reading frame protein	1.43
Q08380	LGALS3BP	Galectin-3-binding protein	1.42
P08648	ITGA5	Integrin alpha-5	1.42
P05534	HLA-A	HLA class I histocompatibility antigen, A-24 alpha chain	1.41
P80303	NUCB2	Nucleobindin-2	1.41
P04083	ANXA1	annexin A1	1.41
Q14980-1	NUMA1	nuclear mitotic apparatus protein 1	1.41
P36957	DLST	Dihydrolipoyllysine-residue succinyltransferase component of 2-oxoglutarate dehydrogenase complex, mitochondrial	1.41
P27105	STOM	erythrocyte band 7 integral membrane protein	1.40
P50454	SERPINH1	Serpin H1	1.40
Q96KB5	PBK	Lymphokine-activated killer T-cell-originated protein kinase	1.40
Q32P28-1	P3H1	Prolyl 3-hydroxylase 1	1.39
Q9Y6A5	TACC3	Transforming acidic coiled-coil-containing protein 3	1.39
Q9NPD8	UBE2T	Ubiquitin-conjugating enzyme E2 T	1.39
P21912	SDHB	Succinate dehydrogenase [ubiquinone] iron-sulfur subunit, mitochondrial	1.39
Q9UHR4	BAIAP2L1	Brain-specific angiogenesis inhibitor 1-associated protein 2-like protein 1	1.39
P06132	UROD	Uroporphyrinogen decarboxylase	1.39
P02765	AHSG	Alpha-2-HS-glycoprotein	1.38

Q9UBS4	DNAJB11	DnaJ homolog subfamily B member 11	1.38
Q15738	NSDHL	sterol-4-alpha-carboxylate 3-dehydrogenase, decarboxylating	1.38
P46926	GNPDA1	glucosamine-6-phosphate isomerase 1	1.38
P01891	HLA-A	HLA class I histocompatibility antigen, A-68 alpha chain	1.38
O60568	PLOD3	Procollagen-lysine,2-oxoglutarate 5-dioxygenase 3	1.37
P50995	ANXA11	annexin A11	1.37
P02794	FTH1	Ferritin heavy chain	1.37
O00762	UBE2C	Ubiquitin-conjugating enzyme E2 C	1.37
Q86X29	LSR	Lipolysis-stimulated lipoprotein receptor	1.37
Q9NYU2-1	UGGT1	UDP-glucose:glycoprotein glucosyltransferase 1	1.37
Q9Y5L4	TIMM13	mitochondrial import inner membrane translocase subunit TIM13	1.37
P21980	TGM2	Protein-glutamine gamma-glutamyltransferase 2	1.36
Q5NDL2	EOGT	EGF domain-specific O-linked N-acetylglucosamine transferase	1.36
P07686	HEXB	Beta-hexosaminidase subunit beta	1.36
P10321	HLA-C	HLA class I histocompatibility antigen, Cw-7 alpha chain	1.36
P19440	GGT1	glutathione hydrolase 1 proenzyme	1.36
P0DI81	TRAPPC2	Trafficking protein particle complex subunit 2	1.36
P14406	COX7A2	Cytochrome c oxidase subunit 7A2, mitochondrial	1.36
Q92878	RAD50	DNA repair protein Rad50	1.35
Q5VW38	GPR107	Protein GPR107	1.35
P20645	M6PR	Cation-dependent mannose-6-phosphate receptor	1.35
O94776	MTA2	Metastasis-associated protein MTA2	1.35
P04062	GBA1	glucosylceramidase	1.34
P13284	IFI30	Gamma-interferon-inducible lysosomal thiol reductase	1.33

P12277	CKB	Creatine kinase B-type	1.33
Q13733	ATP1A4	sodium/potassium-transporting ATPase subunit alpha-4	1.33
Q9H4Z3	PCIF1	Phosphorylated CTD-interacting factor 1	1.32
P06865	HEXA	Beta-hexosaminidase subunit alpha	1.32
Q13509	TUBB3	tubulin beta-3 chain	1.32
Q8TCD1	C18orf32	UPF0729 protein C18orf32	1.32
Q9UHG3	PCYOX1	prenylcysteine oxidase 1	1.32
P09669	COX6C	Cytochrome c oxidase subunit 6C	1.32
P26885	FKBP2	Peptidyl-prolyl cis-trans isomerase FKBP2	1.32
Q9UJX3-1	ANAPC7	Anaphase-promoting complex subunit 7	1.32
P55084	HADHB	Trifunctional enzyme subunit beta, mitochondrial	1.31
P42785-1	PRCP	lysosomal Pro-X carboxypeptidase	1.31
Q5T0N5-1	FNBP1L	Formin-binding protein 1-like	1.31
P05026	ATP1B1	Sodium/potassium-transporting ATPase subunit beta-1	1.31
O00754	MAN2B1	Lysosomal alpha-mannosidase	1.31
P13674-3	P4HA1	Isoform 3 of Prolyl 4-hydroxylase subunit alpha-1	1.31
P00403	MT-CO2	Cytochrome c oxidase subunit 2	1.31
Q8NBJ7-1	SUMF2	Sulfatase-modifying factor 2	1.30
P30484	HLA-B	HLA class I histocompatibility antigen, B-46 alpha chain	1.30
O15118	NPC1	Niemann-Pick C1 protein	1.30
Q9H2V7	SPNS1	Protein spinster homolog 1	1.30
Q9Y276	BCS1L	Mitochondrial chaperone BCS1	1.30
Q9BTU6	PI4K2A	Phosphatidylinositol 4-kinase type 2-alpha	1.30
Q9Y2Q5	LAMTOR2	Ragulator complex protein LAMTOR2	1.30
O14828	SCAMP3	Secretory carrier-associated membrane protein 3	1.30
Q8TAQ2	SMARCC2	SWI/SNF complex subunit SMARCC2	1.30
Q9Y394	DHRS7	Dehydrogenase/reductase SDR family member 7	1.30
Q6NUK1	SLC25A24	Calcium-binding mitochondrial carrier protein SCaMC-1	1.30

P17301	ITGA2	Integrin alpha-2	1.30
Q96C36	PYCR2	Pyrroline-5-carboxylate reductase 2	1.29
Q8WXX5	DNAJC9	dnaJ homolog subfamily C member 9	1.29
P26641	EEF1G	elongation factor 1-gamma	1.29
P00846	MT-ATP6	ATP synthase subunit A	1.29
Q07960	ARHGAP1	rho GTPase-activating protein 1	1.29
Q92597	NDRG1	Protein NDRG1	1.29
P22307-1	SCP2	Non-specific lipid-transfer protein	1.28
P35232	PHB1	Prohibitin	1.28
P11279	LAMP1	Lysosome-associated membrane glycoprotein 1	1.28
P15954	COX7C	Cytochrome c oxidase subunit 7C, mitochondrial	1.28
P14927	UQCRB	Cytochrome b-c1 complex subunit 7	1.28
P46063	RECQL	ATP-dependent DNA helicase Q1	1.28
Q13131	PRKAA1	5'-AMP-activated protein kinase catalytic subunit alpha-1	1.28
O75964	ATP5MG	ATP synthase subunit g, mitochondrial	1.28
P43490	NAMPT	nicotinamide phosphoribosyltransferase	1.27
Q8WTS1	ABHD5	1-acylglycerol-3-phosphate O-acyltransferase ABHD5	1.27
Q9HAV7	GRPEL1	GrpE protein homolog 1, mitochondrial	1.27
Q13671	RIN1	ras and Rab interactor 1	1.27
Q96SQ9	CYP2S1	Cytochrome P450 2S1	1.27
O60264	SMARCA5	SWI/SNF-related matrix-associated actin-dependent regulator of chromatin subfamily A member 5	1.27
P10606	COX5B	Cytochrome c oxidase subunit 5B, mitochondrial	1.27
P09923	ALPI	Intestinal-type alkaline phosphatase	1.26
Q86TX2	ACOT1	acyl-coenzyme A thioesterase 1	1.26
P11441	UBL4A	Ubiquitin-like protein 4A	1.26
O00499	BIN1	Myc box-dependent-interacting protein 1	1.26
P61421	ATP6V0D1	V-type proton ATPase subunit d 1	1.26

Q9P032	NDUFAF4	NADH dehydrogenase [ubiquinone] 1 alpha subcomplex assembly factor 4	1.26
O00534	VWA5A	von Willebrand factor A domain-containing protein 5A	1.26
Q02952-1	AKAP12	A-kinase anchor protein 12	1.26
P23786	CPT2	Carnitine O-palmitoyltransferase 2, mitochondrial	1.26
Q9HCN8	SDF2L1	Stromal cell-derived factor 2-like protein 1	1.26
Q8N2H4	SYS1	protein SYS1 homolog	1.25
P32322	PYCR1	Pyrroline-5-carboxylate reductase 1, mitochondrial	1.25
P49590	HARS2	probable histidine--tRNA ligase, mitochondrial	1.25
O75131	CPNE3	Copine-3	1.25
Q14257	RCN2	Reticulocalbin-2	1.25
P52701	MSH6	DNA mismatch repair protein MSH6	1.25
P25705-1	ATP5F1A	ATP synthase subunit alpha, mitochondrial	1.25
P43121	MCAM	Cell surface glycoprotein MUC18	1.24
Q13309-1	SKP2	S-phase kinase-associated protein 2	1.24
P49189	ALDH9A1	4-trimethylaminobutyraldehyde dehydrogenase	1.24
Q16222-1	UAP1	UDP-N-acetylhexosamine pyrophosphorylase	1.24
Q15067-2	ACOX1	Isoform 2 of Peroxisomal acyl-coenzyme A oxidase 1	1.24
Q9UKK9	NUDT5	ADP-sugar pyrophosphatase	1.24
P06576	ATP5F1B	ATP synthase subunit beta, mitochondrial	1.24
Q3SXM5	HSDL1	Inactive hydroxysteroid dehydrogenase-like protein 1	1.24
O15460-1	P4HA2	Prolyl 4-hydroxylase subunit alpha-2	1.24
O14561	NDUFAB1	Acyl carrier protein, mitochondrial	1.24
Q13423	NNT	NAD(P) transhydrogenase, mitochondrial	1.24
Q09666-1	AHNAK	Neuroblast differentiation-associated protein AHNAK	1.24
O96019-1	ACTL6A	Actin-like protein 6A	1.24

P19474	TRIM21	E3 ubiquitin-protein ligase TRIM21	1.24
Q9UBL3-1	ASH2L	Set1/Ash2 histone methyltransferase complex subunit ASH2	1.24
P31947-1	SFN	14-3-3 protein sigma	1.23
P83916	CBX1	Chromobox protein homolog 1	1.23
P62158	CALM	Calmodulin	1.23
Q16822-1	PCK2	Phosphoenolpyruvate carboxykinase [GTP], mitochondrial	1.23
Q8NBN3-1	TMEM87A	Transmembrane protein 87A	1.23
P08243-1	ASNS	Asparagine synthetase [glutamine-hydrolyzing]	1.23
P50213-1	IDH3A	Isocitrate dehydrogenase [NAD] subunit alpha, mitochondrial	1.23
P31949	S100A11	protein S100-A11	1.23
P40306	PSMB10	Proteasome subunit beta type-10	1.23
O75223-1	GGCT	gamma-glutamylcyclotransferase	1.23
Q6NZI2-1	CAVIN1	caveolae-associated protein 1	1.23
Q9NP97	DYNLRB1	Dynein light chain roadblock-type 1	1.23
Q9UIJ7-1	AK3	GTP:AMP phosphotransferase AK3, mitochondrial	1.22
Q9UNS1	TIMELESS	Protein timeless homolog	1.22
P52789	HK2	Hexokinase-2	1.22
Q86Y82	STX12	Syntaxin-12	1.22
Q96G03	PGM2	Phosphoglucomutase-2	1.22
Q7L266	ASRGL1	isoaspartyl peptidase/L-asparaginase	1.22
Q6RW13	AGTRAP	Type-1 angiotensin II receptor-associated protein	1.22
O14548	COX7A2L	Cytochrome c oxidase subunit 7A-related protein, mitochondrial	1.22
Q9NXV2	KCTD5	BTB/POZ domain-containing protein KCTD5	1.22
P30740	SERPINB1	Leukocyte elastase inhibitor	1.22
P53701	HCCS	Cytochrome c-type heme lyase	1.22
Q9BUI4	POLR3C	DNA-directed RNA polymerase III subunit RPC3	1.22
P40939	HADHA	Trifunctional enzyme subunit alpha, mitochondrial	1.21
Q15758-1	SLC1A5	Neutral amino acid transporter B(0)	1.21

P42126	ECI1	Enoyl-CoA delta isomerase 1, mitochondrial	1.21
P68371	TUBB4B	Tubulin beta-4B chain	1.21
P48047	ATP5PO	ATP synthase subunit O, mitochondrial	1.21
Q9H9B4	SFXN1	Sideroflexin-1	1.21
P05165-1	PCCA	Propionyl-CoA carboxylase alpha chain, mitochondrial	1.21
O95831-1	AIFM1	Apoptosis-inducing factor 1, mitochondrial	1.21
Q9C005	DPY30	Protein dpy-30 homolog	1.21
P09972	ALDOC	Fructose-bisphosphate aldolase C	1.21
O00267-2	SUPT5H	Isoform 2 of Transcription elongation factor SPT5	1.21
P28331-1	NDUFS1	NADH-ubiquinone oxidoreductase 75 kDa subunit, mitochondrial	1.21
O00273-1	DFFA	DNA fragmentation factor subunit alpha	1.21
P12270	TPR	Nucleoprotein TPR	1.21
Q9P0J1	PDP1	[Pyruvate dehydrogenase [acetyl-transferring]]-phosphatase 1, mitochondrial	1.21
Q9UJ70	NAGK	N-acetyl-D-glucosamine kinase	1.21
P00491	PNP	purine nucleoside phosphorylase	1.20
P30154	PPP2R1B	Serine/threonine-protein phosphatase 2A 65 kDa regulatory subunit A beta isoform	1.20
Q04828	AKR1C1	aldo-keto reductase family 1 member C1	1.20
O14530	TXNDC9	Thioredoxin domain-containing protein 9	1.20
Q9Y2R0	COA3	Cytochrome c oxidase assembly factor 3 homolog, mitochondrial	1.20
Q9UK22	FBXO2	F-box only protein 2	1.20
O14787	TNPO2	Transportin-2	1.20
Q8N1G2	CMTR1	Cap-specific mRNA (nucleoside-2'-O-)-methyltransferase 1	1.20
Q92520	FAM3C	Protein FAM3C	1.20
O75947-1	ATP5PD	ATP synthase subunit d, mitochondrial	1.20
O60828	PQBP1	Polyglutamine-binding protein 1	1.20
O43707	ACTN4	Alpha-actinin-4	-1.20

P00533-1	EGFR	epidermal growth factor receptor	-1.20
O43396	TXNL1	Thioredoxin-like protein 1	-1.20
P49915	GMPS	GMP synthase [glutamine-hydrolyzing]	-1.20
P13639	EEF2	Elongation factor 2	-1.21
P30622-2	CLIP1	Isoform 3 of CAP-Gly domain-containing linker protein 1	-1.21
Q96LR5	UBE2E2	Ubiquitin-conjugating enzyme E2 E2	-1.21
Q9Y6U3	SCIN	ADSEVERIN	-1.21
Q00534	CDK6	Cyclin-dependent kinase 6	-1.21
O14920-1	IKBKB	Inhibitor of nuclear factor kappa-B kinase subunit beta	-1.21
O14617-1	AP3D1	AP-3 complex subunit delta-1	-1.21
Q10471	GALNT2	Polypeptide N-acetylgalactosaminyltransferase 2	-1.22
P36507	MAP2K2	Dual specificity mitogen-activated protein kinase kinase 2	-1.22
Q14694	USP10	Ubiquitin carboxyl-terminal hydrolase 10	-1.22
Q00839-2	HNRNPU	Isoform Short of Heterogeneous nuclear ribonucleoprotein U	-1.23
P21291	CSRP1	Cysteine and glycine-rich protein 1	-1.23
P54709	ATP1B3	sodium/potassium-transporting ATPase subunit beta-3	-1.23
Q8NF37	LPCAT1	Lysophosphatidylcholine acyltransferase 1	-1.23
Q14118	DAG1	Dystroglycan	-1.24
P61026	RAB10	ras-related protein rab-10	-1.24
Q16186	ADRM1	Proteasomal ubiquitin receptor ADRM1	-1.24
Q9BTW9	TBCD	Tubulin-specific chaperone D	-1.24
Q96P47	AGAP3	Arf-GAP with GTPase, ANK repeat and PH domain-containing protein 3	-1.25
A3KMH1	VWA8	von Willebrand factor A domain-containing protein 8	-1.25
Q12965	MYO1E	unconventional myosin-Ie	-1.25
Q15366-3	PCBP2	Isoform 3 of Poly(rC)-binding protein 2	-1.25
Q6DKJ4	NXN	nucleoredoxin	-1.25
Q15813	TBCE	Tubulin-specific chaperone E	-1.25
P06748	NPM1	Nucleophosmin	-1.26

Q9ULT8	HECTD1	E3 ubiquitin-protein ligase hectd1	-1.26
Q9Y295	DRG1	developmentally-regulated GTP-binding protein 1	-1.27
P62873	GNB1	Guanine nucleotide-binding protein G(I)/G(S)/G(T) subunit beta-1	-1.27
P51692	STAT5B	Signal transducer and activator of transcription 5B	-1.27
P27816-1	MAP4	Microtubule-associated protein 4	-1.27
Q9H078-2	CLPB	Isoform 2 of Caseinolytic peptidase B protein homolog	-1.27
P59780-1	AP3S2	AP-3 complex subunit sigma-2	-1.27
Q9NR12-1	PDLIM7	PDZ and LIM domain protein 7	-1.28
Q86WA8	LONP2	Lon protease homolog 2, peroxisomal	-1.28
Q9BX95	SGPP1	Sphingosine-1-phosphate phosphatase 1	-1.28
Q0VDF9	HSPA14	Heat shock 70 kDa protein 14	-1.28
P12955	PEPD	xaa-Pro dipeptidase	-1.28
P46777	RPL5	60S ribosomal protein L5	-1.29
Q63HN8-4	RNF213	Isoform 2 of E3 ubiquitin-protein ligase RNF213	-1.29
P22626	HNRNPA2B1	heterogeneous nuclear ribonucleoproteins A2/B1	-1.29
P31942	HNRNPH3	Heterogeneous nuclear ribonucleoprotein H3	-1.30
P25398	RPS12	40S ribosomal protein S12	-1.30
P09884	POLA1	DNA polymerase alpha catalytic subunit	-1.31
Q01105-2	SET	Isoform 2 of Protein SET	-1.31
Q15785	TOMM34	Mitochondrial import receptor subunit TOM34	-1.31
O75534	CSDE1	cold shock domain-containing protein E1	-1.32
Q14444-1	CAPRIN1	Caprin-1	-1.32
Q8NBM4-1	UBAC2	Ubiquitin-associated domain-containing protein 2	-1.32
O60427	FADS1	Fatty acid desaturase 1	-1.32
P32004	L1CAM	Neural cell adhesion molecule L1	-1.32
Q92841	DDX17	Probable ATP-dependent RNA helicase DDX17	-1.32
Q15043	SLC39A14	Zinc transporter ZIP14	-1.32

Q5SSJ5-1	HP1BP3	Heterochromatin protein 1-binding protein 3	-1.33
P68104	EEF1A1	Elongation factor 1-alpha 1	-1.34
P63244	RACK1	Receptor of activated protein C kinase 1	-1.34
Q99615-1	DNAJC7	DnaJ homolog subfamily C member 7	-1.34
P49327	FASN	Fatty acid synthase	-1.35
Q14103-3	HNRNPD	Isoform 3 of Heterogeneous nuclear ribonucleoprotein D0	-1.35
A6NHR9-1	SMCHD1	structural maintenance of chromosomes flexible hinge domain-containing protein 1	-1.35
Q13501-1	SQSTM1	sequestosome-1	-1.35
Q9Y320	TMX2	Thioredoxin-related transmembrane protein 2	-1.35
Q86Y56	DNAAF5	Dynein assembly factor 5, axonemal	-1.37
Q96PK6-1	RBM14	RNA-binding protein 14	-1.37
P61006	RAB8A	Ras-related protein Rab-8A	-1.37
O00571	DDX3X	ATP-dependent RNA helicase DDX3X	-1.37
Q9HBH0	RHOF	Rho-related GTP-binding protein RhoF	-1.38
Q13085-1	ACACA	Acetyl-CoA carboxylase 1	-1.38
Q9BY42	RTF2	protein RTF2 homolog	-1.38
Q9BQG0	MYBBP1A	Myb-binding protein 1A	-1.39
P35221	CTNNA1	Catenin alpha-1	-1.40
P62847-1	RPS24	40S ribosomal protein S24	-1.40
P60866	RPS20	40S ribosomal protein S20	-1.40
O60547-1	GMDS	GDP-mannose 4,6 dehydratase	-1.40
P61254	RPL26	60S ribosomal protein L26	-1.40
Q6PKG0-1	LARP1	La-related protein 1	-1.41
P62899	RPL31	60S ribosomal protein L31	-1.41
P46781	RPS9	40S ribosomal protein S9	-1.42
O60869-1	EDF1	Endothelial differentiation-related factor 1	-1.42
P46782	RPS5	40S ribosomal protein S5	-1.43
P62829	RPL23	60S ribosomal protein L23	-1.44
Q53EL6-1	PDCD4	Programmed cell death protein 4	-1.44
Q9UBF2	COPG2	Coatomer subunit gamma-2	-1.45
P62081	RPS7	40S ribosomal protein S7	-1.46
P62913	RPL11	60S ribosomal protein L11	-1.46

Q9UBB4-1	ATXN10	Ataxin-10	-1.46
P49841-1	GSK3B	Glycogen synthase kinase-3 beta	-1.46
P63173	RPL38	60s ribosomal protein l38	-1.46
P05388	RPLP0	60S acidic ribosomal protein P0	-1.47
P62857	RPS28	40S ribosomal protein S28	-1.47
Q68CZ2-1	TNS3	Tensin-3	-1.50
Q00341-1	HDLBP	Vigilin	-1.50
Q9Y2H5	PLEKHA6	Pleckstrin homology domain-containing family A member 6	-1.50
P46779	RPL28	60S ribosomal protein L28	-1.51
P52735-2	VAV2	Isoform 2 of Guanine nucleotide exchange factor VAV2	-1.51
P62266	RPS23	40S ribosomal protein S23	-1.52
Q93052	LPP	Lipoma-preferred partner	-1.53
P23396-1	RPS3	40S ribosomal protein S3	-1.53
P42677	RPS27	40S ribosomal protein S27	-1.53
P62269	RPS18	40S ribosomal protein S18	-1.54
P61927	RPL37	60S ribosomal protein L37	-1.54
P84098	RPL19	60S ribosomal protein L19	-1.54
O00767	SCD	acyl-CoA desaturase	-1.54
P46776	RPL27A	60S ribosomal protein L27a	-1.55
Q8WX93-1	PALLD	palladin	-1.56
P49207	RPL34	60S ribosomal protein L34	-1.56
P62280	RPS11	40S ribosomal protein S11	-1.56
P32969	RPL9	60S ribosomal protein L9	-1.56
P62277	RPS13	40S ribosomal protein S13	-1.56
P83881	RPL36A	60S ribosomal protein L36a	-1.56
P18621	RPL17	60S ribosomal protein L17	-1.57
Q92522	H1-10	Histone H1x	-1.58
P62854	RPS26	40S ribosomal protein S26	-1.58
P36578	RPL4	60S ribosomal protein L4	-1.58
P62424	RPL7A	60S ribosomal protein L7a	-1.59
Q14244	MAP7	Ensconsin	-1.60
P62888	RPL30	60S ribosomal protein L30	-1.61
Q92625	ANKS1A	Ankyrin repeat and SAM domain-containing protein 1A	-1.61
Q02878	RPL6	60S ribosomal protein L6	-1.62
P61313-1	RPL15	60S ribosomal protein L15	-1.63
P62263	RPS14	40S ribosomal protein S14	-1.64
Q9UNN5-1	FAF1	fas-associated factor 1	-1.64
P39023	RPL3	60S ribosomal protein L3	-1.66
P62841	RPS15	40S ribosomal protein S15	-1.66
P40429	RPL13A	60S ribosomal protein L13a	-1.67

P62241	RPS8	40S ribosomal protein S8	-1.68
P62249	RPS16	40S ribosomal protein S16	-1.71
P50914	RPL14	60S ribosomal protein L14	-1.71
P83731	RPL24	60S ribosomal protein L24	-1.71
P62701	RPS4X	40S ribosomal protein S4, X isoform	-1.72
P15880	RPS2	40S ribosomal protein S2	-1.73
P62906	RPL10A	60S ribosomal protein L10A	-1.73
Q86XZ4	SPATS2	Spermatogenesis-associated serine-rich protein 2	-1.73
P62861	FAU	40S ribosomal protein S30	-1.74
P27635	RPL10	60S ribosomal protein L10	-1.75
P62910	RPL32	60S ribosomal protein L32	-1.76
Q8TDN6	BRIX1	Ribosome biogenesis protein BRX1 homolog	-1.77
Q96NU1	SAMD11	Sterile alpha motif domain-containing protein 11	-1.82
P46783	RPS10	40S ribosomal protein S10	-1.83
P39019	RPS19	40S ribosomal protein S19	-1.86
Q92688	ANP32B	Acidic leucine-rich nuclear phosphoprotein 32 family member B	-1.95
Q7KZF4	SND1	staphylococcal nuclease domain-containing protein 1	-2.00
P62633-1	CNBP	Cellular nucleic acid-binding protein	-2.29

Table S5. MS/MS raw data for protein identification and quantification in A549 cells treated with DDP with $p < 0.05$.

See this table on a separated Excel file named Supporting information Table S5

Table S6. Differentially expressed proteins (DEPs) with $|FC| \geq 1.2$ and p-value <0.05 identified in A549 cells treated with DDP (8.0 μM), in comparison with those in control group of A549 cells.

Accession	gene symbol	Description	fold change
P02647	APOA1	Apolipoprotein A-I	3.21
Q8IUE6	H2AC21	Histone H2A type 2-B	2.35
Q99878	H2AC14	Histone H2A type 1-J	2.29
P35659-1	DEK	Protein DEK	2.28
P62805	H4C1; H4C2; H4C3; H4C4; H4C5; H4C6; H4C8; H4C9; H4C11;	histone H4	2.10

	H4C12; H4C13; H4C14; H4C15; H4C16		
P07305	H1-0	Histone H1.0	1.97
P09874	PARP1	Poly [ADP-ribose] polymerase 1	1.82
P68871	HBB	Hemoglobin subunit beta	1.77
P06396	GSN	Gelsolin	1.69
O95671-1	ASMTL	N-acetylserotonin O-methyltransferase-like protein	1.66
P25815	S100P	Protein S100-P	1.65
P14384	CPM	Carboxypeptidase M	1.65
Q92522	H1-10	Histone H1x	1.64
Q92597	NDRG1	Protein NDRG1	1.61
P08174-1	CD55	Complement decay-accelerating factor	1.56
P42785-1	PRCP	lysosomal Pro-X carboxypeptidase	1.55
P05114	HMGN1	Non-histone chromosomal protein HMG-14	1.53
P11387	TOP1	DNA topoisomerase 1	1.50
P21980	TGM2	Protein-glutamine gamma-glutamyltransferase 2	1.48
P05161	ISG15	Ubiquitin-like protein ISG15	1.47
P05165-1	PCCA	Propionyl-CoA carboxylase alpha chain, mitochondrial	1.45
P09923	ALPI	Intestinal-type alkaline phosphatase	1.45
P31937	HIBADH	3-hydroxyisobutyrate dehydrogenase, mitochondrial	1.44
Q8NDH3	NPEPL1	Probable aminopeptidase NPEPL1	1.44
Q14527	HLTF	Helicase-like transcription factor	1.41
O43852-4	CALU	Isoform 4 of Calumenin	1.41
P17301	ITGA2	Integrin alpha-2	1.40
P02765	AHSG	Alpha-2-HS-glycoprotein	1.40
P14406	COX7A2	Cytochrome c oxidase subunit 7A2, mitochondrial	1.40
P51397	DAP	Death-associated protein 1	1.39
Q13509	TUBB3	tubulin beta-3 chain	1.38
O14773	TPP1	Tripeptidyl-peptidase 1	1.38

O75531	BANF1	Barrier-to-autointegration factor	1.37
Q71UI9-1	H2AZ2	Histone H2A.V	1.37
P21912	SDHB	Succinate dehydrogenase [ubiquinone] iron-sulfur subunit, mitochondrial	1.36
Q14697-1	GANAB	Neutral alpha-glucosidase AB	1.36
P15586	GNS	N-acetylglucosamine-6-sulfatase	1.36
P17096-1	HMGA1	High mobility group protein HMG-I/HMG-Y	1.35
Q8NI22	MCFD2	Multiple coagulation factor deficiency protein 2	1.35
O15173	PGRMC2	Membrane-associated progesterone receptor component 2	1.35
O43852	CALU	Calumenin	1.35
P05787	KRT8	Keratin, type II cytoskeletal 8	1.34
Q16822-1	PCK2	Phosphoenolpyruvate carboxykinase [GTP], mitochondrial	1.34
Q9UBS4	DNAJB11	DnaJ homolog subfamily B member 11	1.34
P17050	NAGA	alpha-N-acetylgalactosaminidase	1.34
P08243-1	ASNS	Asparagine synthetase [glutamine-hydrolyzing]	1.34
Q04695	KRT17	Keratin, type I cytoskeletal 17	1.33
P12074	COX6A1	Cytochrome c oxidase subunit 6A1, mitochondrial	1.33
Q8NBJ7-1	SUMF2	Sulfatase-modifying factor 2	1.33
P06703	S100A6	protein S100-A6	1.33
P27797	CALR	Calreticulin	1.33
Q13510	ASAH1	Acid ceramidase	1.32
Q9Y624	F11R	Junctional adhesion molecule A	1.32
Q9UBF2	COPG2	Coatomer subunit gamma-2	1.32
Q99541	PLIN2	perilipin-2	1.31
P32929	CTH	Cystathionine gamma-lyase	1.31
Q5SSJ5-1	HP1BP3	Heterochromatin protein 1-binding protein 3	1.31

P55789	GFER	FAD-linked sulfhydryl oxidase ALR	1.31
Q96HY7	DHTKD1	Probable 2-oxoglutarate dehydrogenase E1 component DHKTD1, mitochondrial	1.31
Q7L266	ASRGL1	isoaspartyl peptidase/L-asparaginase	1.31
P02749	APOH	Beta-2-glycoprotein 1	1.31
P00403	MT-CO2	Cytochrome c oxidase subunit 2	1.31
Q9BPW8	NIPSNAP1	Protein NipSnap homolog 1	1.30
P28331-1	NDUFS1	NADH-ubiquinone oxidoreductase 75 kDa subunit, mitochondrial	1.30
P05783	KRT18	Keratin, type I cytoskeletal 18	1.30
P18754-1	RCC1	Regulator of chromosome condensation	1.30
Q96FV2	SCRN2	Secernin-2	1.30
P11021	HSPA5	78 kDa glucose-regulated protein	1.30
Q9H0W9	C11orf54	Ester hydrolase C11orf54	1.29
Q9C0B1-1	FTO	alpha-ketoglutarate-dependent dioxygenase FTO	1.29
P16278	GLB1	Beta-galactosidase	1.29
P06737-1	PYGL	Glycogen phosphorylase, liver form	1.29
Q96D15	RCN3	Reticulocalbin-3	1.29
P20020	ATP2B1	Plasma membrane calcium-transporting ATPase 1	1.29
Q13885	TUBB2A	Tubulin beta-2A chain	1.29
Q86Y82	STX12	Syntaxin-12	1.29
P56199	ITGA1	Integrin alpha-1	1.29
Q04828	AKR1C1	aldo-keto reductase family 1 member C1	1.29
P05166-1	PCCB	propionyl-CoA carboxylase beta chain, mitochondrial	1.28
Q9BZQ8	NIBAN1	Protein Niban	1.28
P08648	ITGA5	Integrin alpha-5	1.28
Q13451-1	FKBP5	Peptidyl-prolyl cis-trans isomerase FKBP5	1.28
P07686	HEXB	Beta-hexosaminidase subunit beta	1.28

Q9NXV2	KCTD5	BTB/POZ domain-containing protein KCTD5	1.28
Q12888-1	TP53BP1	TP53-binding protein 1	1.28
O96019-1	ACTL6A	Actin-like protein 6A	1.28
P36405	ARL3	ADP-ribosylation factor-like protein 3	1.27
Q9BQI0	AIF1L	allograft inflammatory factor 1-like	1.27
Q03405	PLAUR	Urokinase plasminogen activator surface receptor	1.27
Q9NWM8	FKBP14	Peptidyl-prolyl cis-trans isomerase FKBP14	1.27
Q9UHR4	BAIAP2L1	Brain-specific angiogenesis inhibitor 1-associated protein 2-like protein 1	1.27
Q9Y6C9	MTCH2	Mitochondrial carrier homolog 2	1.26
O60264	SMARCA5	SWI/SNF-related matrix-associated actin-dependent regulator of chromatin subfamily A member 5	1.26
Q14914	PTGR1	Prostaglandin reductase 1	1.26
Q16740	CLPP	ATP-dependent Clp protease proteolytic subunit, mitochondrial	1.26
O95825-1	CRYZL1	Quinone oxidoreductase-like protein 1	1.26
Q9UNW1	MINPP1	Multiple inositol polyphosphate phosphatase 1	1.26
Q9NYU2-1	UGGT1	UDP-glucose:glycoprotein glucosyltransferase 1	1.26
P17174	GOT1	Aspartate aminotransferase, cytoplasmic	1.26
Q9BWM7	SFXN3	Sideroflexin-3	1.26
Q8IWL3	HSCB	iron-sulfur cluster co-chaperone protein HscB, mitochondrial	1.26
Q02252	ALDH6A1	Methylmalonate-semialdehyde dehydrogenase [acylating], mitochondrial	1.26
Q9UBG0	MRC2	C-type mannose receptor 2	1.26
Q13162	PRDX4	Peroxiredoxin-4	1.25

Q9Y2Q5	LAMTOR2	Ragulator complex protein LAMTOR2	1.25
P03905	MT-ND4	NADH-ubiquinone oxidoreductase chain 4	1.25
P09525	ANXA4	annexin A4	1.25
P07203	GPX1	Glutathione peroxidase 1	1.25
P00367	GLUD1	Glutamate dehydrogenase 1, mitochondrial	1.25
P23219	PTGS1	Prostaglandin G/H synthase 1	1.25
Q9H4Z3	PCIF1	Phosphorylated CTD-interacting factor 1	1.25
Q8WZA0	LZIC	Protein LZIC	1.24
O15126-1	SCAMP1	Secretory carrier-associated membrane protein 1	1.24
Q9P0J1	PDP1	[Pyruvate dehydrogenase [acetyl-transferring]]-phosphatase 1, mitochondrial	1.24
P32322	PYCR1	Pyrroline-5-carboxylate reductase 1, mitochondrial	1.24
Q9HDC9	APMAP	Adipocyte plasma membrane-associated protein	1.23
P30043	BLVRB	flavin reductase (NADPH)	1.23
Q6PIU2	NCEH1	Neutral cholesterol ester hydrolase 1	1.23
O75251	NDUFS7	NADH dehydrogenase [ubiquinone] iron-sulfur protein 7, mitochondrial	1.23
Q7Z2K6	ERMP1	Endoplasmic reticulum metallopeptidase 1	1.23
P13284	IFI30	Gamma-interferon-inducible lysosomal thiol reductase	1.23
P16083	NQO2	Ribosyldihydronicotinamide dehydrogenase [quinone]	1.23
P31327-1	CPS1	Carbamoyl-phosphate synthase [ammonia], mitochondrial	1.23
Q6NVY1	HIBCH	3-hydroxyisobutyryl-CoA hydrolase, mitochondrial	1.23
Q92882	OSTF1	osteoclast-stimulating factor 1	1.23
P14209	CD99	CD99 antigen	1.23

P09669	COX6C	Cytochrome c oxidase subunit 6C	1.23
P09960-1	LTA4H	leukotriene A-4 hydrolase	1.23
P31150	GDI1	Rab GDP dissociation inhibitor alpha	1.23
O15460-1	P4HA2	Prolyl 4-hydroxylase subunit alpha-2	1.23
P20340-2	RAB6A	Isoform 2 of Ras-related protein Rab-6A	1.23
Q9UIJ7-1	AK3	GTP:AMP phosphotransferase AK3, mitochondrial	1.23
P52701	MSH6	DNA mismatch repair protein MSH6	1.23
P06865	HEXA	Beta-hexosaminidase subunit alpha	1.23
Q13423	NNT	NAD(P) transhydrogenase, mitochondrial	1.23
Q9UJS0	SLC25A13	Calcium-binding mitochondrial carrier protein Aralar2	1.22
Q14651	PLS1	Plastin-1	1.22
P15374	UCHL3	Ubiquitin carboxyl-terminal hydrolase isozyme L3	1.22
O95197-3	RTN3	Isoform 3 of Reticulon-3	1.22
Q13492-1	PICALM	Phosphatidylinositol-binding clathrin assembly protein	1.22
Q969H8	MYDGF	Myeloid-derived growth factor	1.22
P35754	GLRX	Glutaredoxin-1	1.22
Q9H488-1	POFUT1	GDP-fucose protein O-fucosyltransferase 1	1.22
Q5T0N5-1	FNBP1L	Formin-binding protein 1-like	1.22
Q86X76	NIT1	Deaminated glutathione amidase	1.22
O94888	UBXN7	UBX domain-containing protein 7	1.22
Q96DZ1	ERLEC1	Endoplasmic reticulum lectin 1	1.22
P30040-1	ERP29	Endoplasmic reticulum resident protein 29	1.22

P27338	MAOB	Amine oxidase [flavin-containing] B	1.22
Q9BS26	ERP44	Endoplasmic reticulum resident protein 44	1.22
P10696	ALPG	Alkaline phosphatase, placental-like	1.22
P23634-1	ATP2B4	Plasma membrane calcium-transporting ATPase 4	1.22
Q96CP2	FLYWCH2	FLYWCH family member 2	1.22
O43169	CYB5B	Cytochrome b5 type B	1.22
P50995	ANXA11	annexin A11	1.21
Q8WTS1	ABHD5	1-acylglycerol-3-phosphate O-acyltransferase ABHD5	1.21
P19404	NDUFV2	NADH dehydrogenase [ubiquinone] flavoprotein 2, mitochondrial	1.21
P36543	ATP6V1E1	V-type proton ATPase subunit E 1	1.21
Q6P4Q7	CNNM4	metal transporter CNNM4	1.21
Q8TC07-3	TBC1D15	Isoform 3 of TBC1 domain family member 15	1.21
O75323-1	NIPSNAP2	Protein NipSnap homolog 2	1.21
P54577	YARS1	Tyrosine--tRNA ligase, cytoplasmic	1.21
Q9UHG3	PCYOX1	prenylcysteine oxidase 1	1.21
P46108-1	CRK	Adapter molecule crk	1.21
Q9BRK5-1	SDF4	45 kDa calcium-binding protein	1.21
P61916	NPC2	Epididymal secretory protein E1	1.21
Q13642-1	FHL1	Isoform 1 of Four and a half LIM domains protein 1	1.21
O43598-1	DNPH1	2'-deoxynucleoside 5'-phosphate N-hydrolase 1	1.21
Q9ULU4	ZMYND8	protein kinase C-binding protein 1	1.21
Q9GZQ8	MAP1LC3B	Microtubule-associated proteins 1A/1B light chain 3B	1.21
Q9H0E2	TOLLIP	Toll-interacting protein	1.21
P20073-1	ANXA7	Annexin A7	1.20
Q16795	NDUFA9	NADH dehydrogenase [ubiquinone] 1 alpha	1.20

		subcomplex subunit 9, mitochondrial	
Q9Y277	VDAC3	Voltage-dependent anion-selective channel protein 3	1.20
P13929-1	ENO3	Beta-enolase	1.20
P11498	PC	pyruvate carboxylase, mitochondrial	1.20
P23368-1	ME2	NAD-dependent malic enzyme, mitochondrial	1.20
P29373	CRABP2	Cellular retinoic acid-binding protein 2	1.20
O95571	ETHE1	Persulfide dioxygenase ETHE1, mitochondrial	1.20
O00754	MAN2B1	Lysosomal alpha-mannosidase	1.20
P60842	EIF4A1	Eukaryotic initiation factor 4A-I	-1.21
P27105	STOM	erythrocyte band 7 integral membrane protein	-1.21
P61289-1	PSME3	Proteasome activator complex subunit 3	-1.21
Q8TEX9-1	IPO4	Importin-4	-1.21
P62249	RPS16	40S ribosomal protein S16	-1.21
P10321	HLA-C	HLA class I histocompatibility antigen, Cw-7 alpha chain	-1.21
P11142-1	HSPA8	Heat shock cognate 71 kDa protein	-1.21
Q03013-1	GSTM4	glutathione S-transferase mu 4	-1.21
P62701	RPS4X	40S ribosomal protein S4, X isoform	-1.21
P33552	CKS2	cyclin-dependent kinases regulatory subunit 2	-1.21
P36578	RPL4	60S ribosomal protein L4	-1.22
P24534	EEF1B2	Elongation factor 1-beta	-1.22
P47813	EIF1AX	Eukaryotic translation initiation factor 1A, X-chromosomal	-1.22
Q92688	ANP32B	Acidic leucine-rich nuclear phosphoprotein 32 family member B	-1.22
Q9BQG0	MYBBP1A	Myb-binding protein 1A	-1.22

P24928	POLR2A	DNA-directed RNA polymerase II subunit RPB1	-1.22
O00214-1	LGALS8	Galectin-8	-1.22
P61006	RAB8A	Ras-related protein Rab-8A	-1.22
Q9Y672	ALG6	Dolichyl pyrophosphate Man9GlcNAc2 alpha-1,3-glucosyltransferase	-1.22
P49327	FASN	Fatty acid synthase	-1.23
P41091	EIF2S3	eukaryotic translation initiation factor 2 subunit 3	-1.23
P26374	CHML	Rab proteins geranylgeranyltransferase component A 2	-1.23
Q09666-1	AHNAK	Neuroblast differentiation-associated protein AHNAK	-1.23
P61978-2	HNRNPK	Isoform 2 of Heterogeneous nuclear ribonucleoprotein K	-1.24
P46781	RPS9	40S ribosomal protein S9	-1.24
P30408	TM4SF1	Transmembrane 4 L6 family member 1	-1.24
P50454	SERPINH1	Serpin H1	-1.25
P26641	EEF1G	elongation factor 1-gamma	-1.25
Q9BV57	ADI1	1,2-dihydroxy-3-keto-5-methylthiopentene dioxygenase	-1.25
P35221	CTNNA1	Catenin alpha-1	-1.26
P39019	RPS19	40S ribosomal protein S19	-1.26
Q13501-1	SQSTM1	sequestosome-1	-1.26
Q53FV1	ORMDL2	ORM1-like protein 2	-1.26
Q9NRW3	APOBEC3C	DNA dC->dU-editing enzyme APOBEC-3C	-1.27
Q99615-1	DNAJC7	DnaJ homolog subfamily C member 7	-1.27
Q9Y320	TMX2	Thioredoxin-related transmembrane protein 2	-1.27
P54709	ATP1B3	sodium/potassium-transporting ATPase subunit beta-3	-1.28
O00762	UBE2C	Ubiquitin-conjugating enzyme E2 C	-1.29
Q14444-1	CAPRIN1	Caprin-1	-1.30
P15880	RPS2	40S ribosomal protein S2	-1.30
Q9NRX1	PNO1	RNA-binding protein pno1	-1.30

O76021	RSL1D1	Ribosomal L1 domain-containing protein 1	-1.30
P50238	CRIP1	Cysteine-rich protein 1	-1.30
Q9NR30-1	DDX21	Nucleolar RNA helicase 2	-1.30
P17812	CTPS1	CTP synthase 1	-1.30
Q9Y6M0-1	PRSS21	Testisin	-1.30
Q9Y2H5	PLEKHA6	Pleckstrin homology domain-containing family A member 6	-1.30
Q8WTV0-2	SCARB1	Isoform 1 of Scavenger receptor class B member 1	-1.30
P53999	SUB1	Activated RNA polymerase II transcriptional coactivator p15	-1.31
P42771-1	CDKN2A	cyclin-dependent kinase inhibitor 2A	-1.31
Q00534	CDK6	Cyclin-dependent kinase 6	-1.31
Q9UQ35	SRRM2	serine/arginine repetitive matrix protein 2	-1.31
Q9UG63	ABCF2	ATP-binding cassette sub-family F member 2	-1.33
Q9NY93	DDX56	Probable ATP-dependent RNA helicase DDX56	-1.33
Q8WUX1-1	SLC38A5	Sodium-coupled neutral amino acid transporter 5	-1.34
O95816	BAG2	BAG family molecular chaperone regulator 2	-1.35
O15234	CASC3	Protein CASC3	-1.35
P12277	CKB	Creatine kinase B-type	-1.36
P31689-1	DNAJA1	DnaJ homolog subfamily A member 1	-1.37
Q16850-1	CYP51A1	lanosterol 14-alpha demethylase	-1.37
P28288	ABCD3	ATP-binding cassette sub-family D member 3	-1.37
Q9H2J4	PDCL3	phosducin-like protein 3	-1.37
O75534	CSDE1	cold shock domain-containing protein E1	-1.38
Q9P275	USP36	Ubiquitin carboxyl-terminal hydrolase 36	-1.39
O43688	PLPP2	Phospholipid phosphatase 2	-1.39

O95433	AHSA1	activator of 90 kDa heat shock protein ATPase homolog 1	-1.39
Q15043	SLC39A14	Zinc transporter ZIP14	-1.39
Q9BY42	RTF2	protein RTF2 homolog	-1.40
P08962	CD63	CD63 antigen	-1.41
Q9BZX2-1	UCK2	Uridine-cytidine kinase 2	-1.41
Q9Y3C8	UFC1	ubiquitin-fold modifier-conjugating enzyme 1	-1.41
P68104	EEF1A1	Elongation factor 1-alpha 1	-1.42
P23921	RRM1	Ribonucleoside-diphosphate reductase large subunit	-1.42
P17813	ENG	Endoglin	-1.49
O00767	SCD	acyl-CoA desaturase	-1.51
P04792	HSPB1	Heat shock protein beta-1	-1.51
Q6ICB0	DESI1	Desumoylating isopeptidase 1	-1.51
P17844	DDX5	probable ATP-dependent RNA helicase DDX5	-1.54
Q01581	HMGCS1	hydroxymethylglutaryl-CoA synthase, cytoplasmic	-1.70

Table S7. MS/MS raw data for identification and quantification of histone acetylation level in A549 cells treated with DDP in the absence or the absence of TSA

See this table on a separated Excel file named Supporting information Table S7

Table S8. Histone acetylation level identified and quantified in A549 cells treated with DDP (8 μM) and TSA (1 μM), in comparison with those in A549 cells treat with DDP (8 μM) alone.

Histone	Site	Flod Change	p-value
Histone H2A	Lysine 5	3.77	0.35
Histone H2A	Lysine 7	2.50	0.17
Histone H2B	Lysine 11	2.48	0.07
Histone H2B	Lysine 12	3.33	0.24
Histone H2B	Lysine 15	3.33	0.24
Histone H2B	Lysine 16	3.33	0.24
Histone H2B	Lysine 20	2.08	0.04
Histone H3	Lysine 14	1.98	0.34
Histone H3	Lysine 18	3.01	0.16
Histone H3	Lysine 23	1.76	0.33
Histone H3	Lysine 27	2.48	0.08

Histone H4	Lysine 5	3.76	0.16
Histone H4	Lysine 8	3.07	0.05
Histone H4	Lysine 12	1.56	0.21
Histone H4	Lysine 16	1.56	0.21
